# Enantiomeric diarylheptanoids from *Ottelia* *acuminata* var. *acuminata* and their *α*-glucosidase inhibitory activity

**DOI:** 10.1007/s13659-025-00515-w

**Published:** 2025-05-16

**Authors:** Jia-Ru Zhou, Xin-Yue Hu, Hong-Xing Liu, Yu Zhou, Fei-Fei Xiong, Jian-Jun Zhao, Xing-Ren Li, Gang Xu

**Affiliations:** 1 State Key Laboratory of Phytochemistry and Natural Medicines, and Yunnan Key Laboratory of Natural Medicinal Chemistry, Kunming Institute of Botany, Chinese Academy of Sciences, Kunming, 650201 China; 2 University of Chinese Academy of Sciences, Beijing, 100049 China

**Keywords:** *Otteliaacuminata*, Diarylheptanoids, Enantiomers, *α*-Glucosidase inhibitory activity

## Abstract

Otteacumienes G–K (**1**–**5**), five pairs of enantiomeric diarylheptanoids, along with one undescribed diarylheptanoid glycoside and one new lignan, were isolated from *Ottelia* *acuminata* var. *acuminata*. Compounds **1**–**5** were identified as five pairs of enantiomers and their structural configurations were determined through a combination of spectroscopic analysis, X-ray crystallography, and ECD calculation. Notably, compound **6** was the first diarylheptanoid glycoside isolated from this aquatic species, and its absolute configuration was unequivocally established through semi-synthesis. Biological evaluation demonstrated that compound **1** exhibited *α*-glucosidase inhibitory activity, with an inhibition ratio of 38.97% (acarbose as the positive control, inhibition ratio = 13.52%).

**Graphical abstract:**

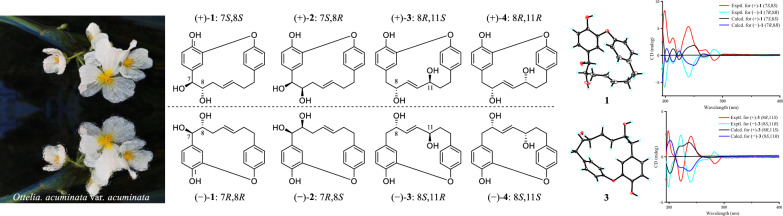

**Supplementary Information:**

The online version contains supplementary material available at 10.1007/s13659-025-00515-w.

## Introduction

Diabetes mellitus (DM) represents a heterogeneous group of metabolic disorders characterized by chronic hyperglycemia, clinically categorized as type 1 (T1D) and type 2 (T2D), specific types of diabetes and gestational diabetes [1]. Among these, T2D, the most common type, is characterized by a progressive loss of insulin secretion heterogeneity in pancreatic *β*-cells, typically following the development of insulin resistance [2]. There is no definitive cure for T2D, but its progression can be effectively managed through a combination of lifestyle modifications and pharmacological interventions [3]. Current therapeutic strategies primarily involve the use of insulin and its analogs, biguanides (e.g., metformin), sodium-glucose cotransporter 2 (SGLT2) inhibitors, *α*-glucosidase inhibitors (AGIs), glucagon-like peptide-1 **(**GLP-1) receptor agonists, and dipeptidyl peptidase 4 (DPP-4) inhibitors [2].

*α*-Glucosidase is a key enzyme in carbohydrate metabolism responsible for catalyzing the final step of dietary carbohydrate digestion [3]. The therapeutic potential of AGIs in diabetes management has been well established, particularly for controlling postprandial hyperglycemia and preventing associated complications in T2D patients [4]. However, the commonly used AGIs at present are frequently associated with gastrointestinal adverse effects such as diarrhea, bloating, and nausea, which significantly hinder their clinical utilization and widespread adoption [5]. These limitations underscore the critical need for developing novel AGIs with improved safety profiles and enhanced therapeutic efficacy.

*Ottelia* *acuminata* var. *acuminata*, a perennial herbaceous species of the *Ottelia* genus in the Hydrocharitaceae family, is predominantly distributed in the provinces of Guangdong, Guangxi, Guizhou, and Yunnan [6]. In the traditional Chinese medicine system, this species has been widely employed for the treatment of constipation, bronchitis, and arteriosclerosis [7].

**Fig.1:**
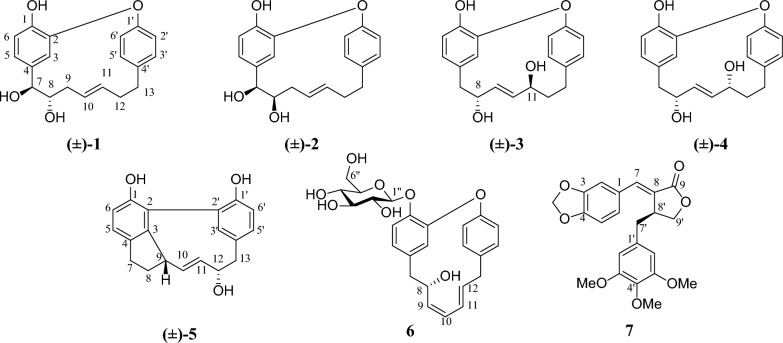
The chemical structures of compounds **1**–**7**

Previous phytochemical investigations of this plant have revealed the presence of diverse chemical constituents including diarylheptanoids, lignans, coumarins, flavonoids, and sesquiterpenes [8]. Notably, diarylheptanoids exhibited significant hypoglycemic potential by inhibiting *α*-glucosidase [8]. As part of our ongoing research to discover hypoglycemic lead compounds, five pairs of enantiomeric diarylheptanoids, one undescribed diarylheptanoid glycoside, and one new lignan were isolated from *O.* *acuminata* (Fig. 1). In this study, compounds **1**–**5** were identified as racemates and the absolute configurations of **1**–**4** were determined by combination of NMR, ECD spectroscopy, and X-ray diffraction analysis. In addition, compounds **1**, **2**,** 4**, **6**, and** 7** were evaluated for their inhibitory activities against *α*-glucosidase and protein tyrosine phosphatase 1B (PTP1B). Among these, compound **1** exhibited notable *α*-glucosidase inhibitory activity, with an inhibition ratio of 38.97% (acarbose as the positive control, inhibition ratio = 13.52%).

## Result and discussion

**Fig. 2:**
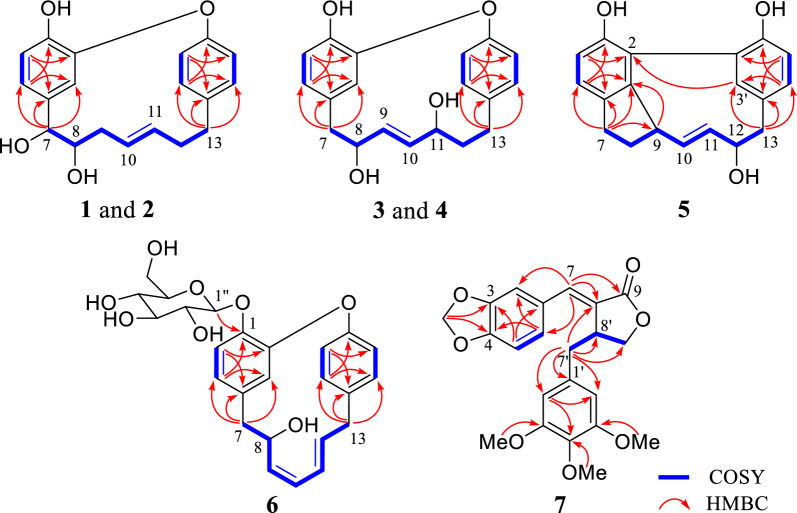
Key ^1^H–^1^H COSY and HMBC correlations of **1**–**7**

Otteacumiene G (**1**), isolated as colorless crystals, was assigned the molecular formula of C_19_H_20_O_4_ by the HRESIMS at *m/z* 311.1287 [M − H]^−^ (calculated for 311.1289), suggesting ten degrees of unsaturation. The IR spectrum showed a broad hydroxyl absorption band at 3426 cm^−1^ and aromatic ring absorption band at 1613, 1596, 1516, 1502 cm^−1^. The ^13^C NMR (DEPT) spectra (Table 2) exhibited 19 carbon signals, including two benzene rings (*δ*_C_ 146.6, C-1; 151.3, C-2; 118.4, C-3; 134.5, C-4; 120.7, C-5; 117.0, C-6 and 158.0, C-1′; 123.7, C-2′; 133.5, C-3′; 140.7, C-4′; 131.3, C-5′; 124.8, C-6′) and an oxygenated unsaturated heptane (*δ*_C_ 76.3, C-7; 76.9, C-8; 36.3, C-9; 128.9, C-10; 132.0, C-11; 37.1, C-12; 35.9, C-13). The key HMBC correlations from H-7 to C-3/C-4/C-5 and H_2_-13 to C-3′/C-4′/C-5′ suggested **1** was a diarylheptanoid derivative. By combining HRESIMS and comparing NMR (Tables 1 and 2), compound **1** was identified to share the same diarylether-type cyclic diarylheptanoid skeleton as the known otteacumiene C [7], with only variation occurring in the heptane chain, a hydroxyl group at C-7 (*δ*_C_ 76.3). Furthermore, this deduction was confirmed by key ^1^H-^1^H COSY correlations of H-7 (*δ*_H_ 4.33, d, *J* = 6.8 Hz)/H-8 (*δ*_H_ 3.34, m)/H_2_-9/H-10 (*δ*_H_ 4.84, overlapped)/H-11 (*δ*_H_ 5.15, dt, *J* = 15.3, 6.1 Hz) /H_2_-12/H_2_-13 (Fig. 2). Besides, the ^1^H NMR signals at *δ*_H_ 4.84 (H-10) and *δ*_H_ 5.15 (H-11) also suggested the existence of a *trans* double bond [9]. Consequently, the planar structure of **1** as a diarylether-type cyclic diarylheptanoid was unequivocally determined.

**Table 1:** ^1^H NMR data of compounds **1**–**5** in CD_3_OD

No.	**1**	**2**	**3**	**4**	**5**
3	5.61, d (1.7)	5.63, d, (1.4)	5.66, d (1.8)	5.65, d (2.0)	
5	6.82, dd (8.2, 1.7)	6.84, dd (8.2, 1.4)	6.55, dd (8.1, 1.8)	6.54, dd (8.2, 2.0)	7.06, d (8.0)
6	6.79, d (8.2)	6.80, d (8.2)	6.69, d (8.1)	6.71, d (8.2)	6.72, d (8.0)
7a	4.33, d (6.8)	4.49, d (2.5)	2.71, dd (13.8, 3.1)	2.66, dd (15.2, 8.4)	2.86, m
7b			2.44, dd (13.8, 9.5)	2.53, dd (15.2, 2.0)	2.72, m
8a	3.34, m	3.64, m	3.99, m	3.82, td (8.2, 2.1)	2.22, m
8b					1.98, m
9a	1.98, m	1.98, m	5.16, dd (15.8, 4.7)	5.26, dd (15.4, 8.2)	3.35, m
9b	1.72, d (15.8)	1.68, m			
10	4.84, overlapped	4.78, dt (15.3, 5.7)	5.51, dd (15.8, 7.2)	5.32, dd (15.4, 8.2)	4.06, dd (17.1, 7.1)
11	5.15, dt (15.3, 6.1)	5.15, dt (15.3, 7.5)	4.04, m	3.93, td (7.4, 1.7)	5.36, dd (17.1, 7.6)
12a	2.08, m	2.41, m	2.05, m	2.03, m	3.81, m
12b	2.39, m	2.06, m	1.74, m	1.93, m	
13a	2.88, dt (12.6, 5.0)	2.90, m	2.65, m	2.68, m	2.49, t (12.4)
13b	2.66, m	2.66, m	2.99, dt (13.3, 4.4)	3.04, dt (13.6, 4.1)	3.10, dd (12.4, 5.0)
2′	6.83, dd (8.3, 2.3)	6.85, dd (8.3, 2.3)	6.95, dd (8.3, 2.5)	6.94, dd (8.2, 2.5)	
3′	6.98, dd (8.3, 1.9)	7.00, dd (8.3, 1.8)	7.31, dd (8.3, 1.9)	7.25, dd (8.2, 2.0)	6.38, d (2.0)
5′	7.14, dd (8.3, 1.9)	7.15, dd (8.3, 1.8)	7.32, dd (8.3, 1.9)	7.35, dd (8.2, 2.0)	7.02, dd (8.0, 2.0)
6′	7.09, dd (8.3, 2.3)	7.09, dd (8.3, 2.3)	7.09, dd (8.3, 2.5)	7.00, dd (8.2, 2.5)	6.78, d (8.0)

*δ* in ppm, *J* in Hz, and obtained at 600 MHz

**Table 2:** ^13^C NMR and DEPT data of compounds **1**–**5** in CD_3_OD

No.	**1**	**2**	**3**	**4**	**5**
1	146.6, s	146.4, s	145.3, s	145.2, s	153.7, s
2	151.3, s	150.9, s	151.0, s	151.1, s	125.3, s
3	118.4, d	117.8, d	118.7, d	117.2, d	149.9, s
4	134.5, s	133.0, s	130.2, s	130.7, s	137.0, s
5	120.7, d	122.4, d	123.8, d	123.8, d	125.5, d
6	117.0, d	117.0, d	116.7, d	116.9, d	114.3, d
7	76.3, d	76.3, d	43.5, t	42.7, t	31.4, t
8	76.9, d	75.0, d	72.3, d	74.0, d	32.4, t
9	36.3, t	36.1, t	134.7, d	137.1, d	48.7, d
10	128.9, d	129.6, d	134.9, d	139.4, d	137.3, d
11	132.0, d	131.6, d	72.4, d	73.1, d	129.2, d
12	37.1, t	36.8, t	41.5, t	40.9, t	79.9, d
13	35.9, t	35.8, t	33.5, t	34.0, t	47.0, t
1′	158.0, s	157.4, s	157.0, s	156.7, s	154.9, s
2′	123.7, d	123.5, d	123.5, d	124.4, d	120.8, s
3′	133.5, d	133.9, d	132.4, d	132.3, d	145.9, d
4′	140.7, s	140.7, s	141.1, s	140.2, s	129.4, s
5′	131.3, d	131.2, d	131.4, d	131.5, d	129.6, d
6′	124.8, d	124.8, d	125.9, d	125.1 d	115.9, d

*δ* in ppm and obtained at 150 MHz

**Fig. 3:**
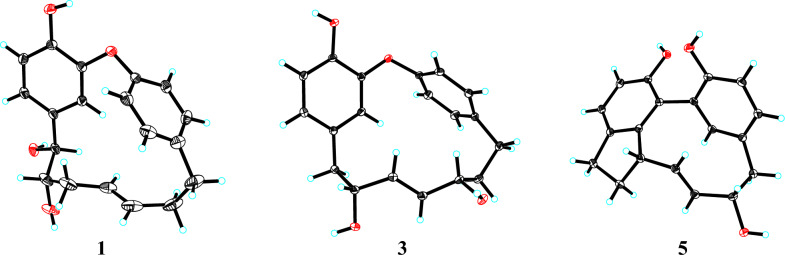
X-ray crystallographic structures for **1**, **3** and **5**

**Fig. 4:**
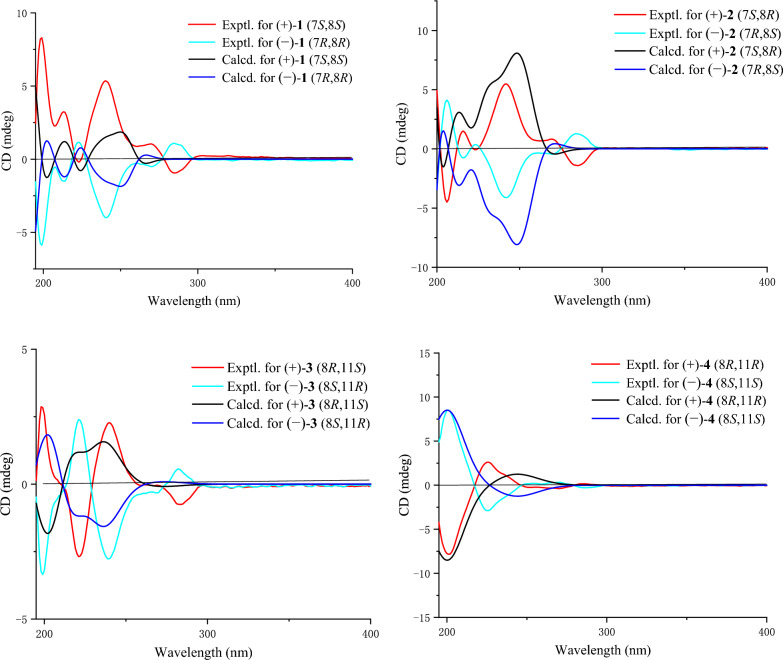
Comparison of experimental and calculated ECD spectra of compounds **1**–**4**

The cotton effects were not observed in the ECD spectrum of **1** and it was separated into two peaks by chiral-phase column (Fig. S1). Thus, **1** was a racemic mixture [10]. Fortunately, the crystals of **1** (Fig. 3) were obtained, which showed the relative configuration of **1**. Following the chiral resolution of this compound, the absolute configurations of the resultant (+)-**1** and (−)-**1** enantiomers were unequivocally established via quantum chemical calculation. The results demonstrated that the calculated ECD spectra of the enantiomeric pair exhibited excellent agreement with the experimental data, allowing unambiguous assignment of the absolute configurations as 7*S*,8*S* for (+)-**1** and 7*R*,8*R* for (−)-**1** (Fig. 4).

Otteacumiene H (**2**), isolated as colorless crystals, had its molecular formula C_19_H_20_O_4_, which was evidenced by the HRESIMS at *m/z* 311.1287 [M − H]^−^ (calculated for 311.1289). Compound **2** shared the same planar structure as **1,** as established by the detailed ^1^H-^1^H COSY and HMBC correlations (Fig. 2). The *trans*-configuration of the double bond was confirmed by the coupling constant of the olefinic proton *δ*_H_ 5.15 (1H, dt, *J* = 15.3, 7.5 Hz, H-11). Comparative analysis of the ^1^H and ^13^C NMR data (Tables 1 and 2) revealed a key difference at the chiral center C-8 (*δ*_H_ 3.64, m, *δ*_C_ 75.0 in **2**; *δ*_H_ 3.34, m, *δ*_C_ 76.9 in **1**), suggesting that **2** was a stereoisomer of **1**. As the vicinal dihydroxyl groups in compound **1** adopt a *trans* configuration, the corresponding groups in compound **2** were *cis*-configured. With only two chiral centers (C-7 and C-8) in this planar structure, there existed two relative configurations and two possible absolute configurations, excluding the already established 7*S*,8*S* and 7*R*,8*R* configurations of (+)-**1** and (−)-**1**, respectively. Further analysis of the ECD spectrum and chiral HPLC data (Fig. S14) confirmed that compound **2** existed as a racemate. Through chiral resolution followed by comparison of experimental and calculated ECD curves, the absolute configurations of (+)-**2** and (−)-**2** were assigned as 7*S*,8*R* and 7*R*,8*S*, respectively (Fig. 4).

Otteacumiene I (**3**) was purified as colorless crystals. Its molecular formula was determined as C_19_H_20_O_4_ by its HRESIMS (*m/z* 311.1286, [M − H]^−^, calculated for 311.1289). A detailed comparison of the HRESIMS and NMR data (Tables 1 and 2) of **3** with those of **1** revealed that they were close structural analogs. The differences from **1** were the positional variations of hydroxyl groups (*δ*_C_ 72.4, C-11) and double bond (*δ*_C_ 134.7, C-9; 134.9, C-10) in **3**, as confirmed by the key ^1^H-^1^H COSY correlations of H_2_-7 (*δ*_H_ 2.71, dd, *J* = 13.8, 3.1 Hz; 2.44, dd, *J* = 13.8, 9.5 Hz)/H-8 (*δ*_H_ 3.99, m) /H-9 (*δ*_H_ 5.16, dd, *J* = 15.8, 4.7 Hz)/H-10 (*δ*_H_ 5.51, dd, *J* = 15.8, 7.2 Hz)/H-11 (*δ*_H_ 4.04, m)/H_2_-12/H_2_-13 and HMBC correlations from H-7 to C-3 (*δ*_C_ 118.7), C-4 (*δ*_C_ 130.2), and C-5 (*δ*_C_ 123.8); from H_2_-13 to C-3′ (*δ*_C_ 132.4), C-4′ (*δ*_C_ 141.1), and C-5′ (*δ*_C_ 131.4) (Fig. 2).

The appearance of two peaks on chiral-phase column (Fig. S27) and the absence of an obvious cotton effect in the ECD spectrum indicated that compound **3** was a racemate. Furthermore, the relative configurations of **3** were proposed by the result of single-crystal X-ray diffraction (Fig. 3). After chiral separation of (±)-**3**, the absolute configurations of (+)-**3** and (−)-**3** were determined as 8*R*,11*S* and 8*S*,11*R* by comparing the experimental and calculated ECD curves (Fig. 4).

Otteacumiene J (**4**), a racemic mixture (Fig. S40), was purified as white powder. It had the same molecular formula as compound **3**, as deduced by HRESIMS (*m/z* 311.1285, [M − H]^−^, calculated for 311.1289). Comparative analyses of ^1^H and ^13^C NMR data (Tables 1 and 2) between **3** and **4** revealed that **4** was an epimer of **3**, which was supported by the changes in chemical shift of C-7 (*δ*_C_ 42.7), C-8 (*δ*_C_ 74.0), C-9 (*δ*_C_ 137.1), C-10 (*δ*_C_ 139.4), and C-11 (*δ*_C_ 73.1). The relative configurations of **3** were proposed by the result of single-crystal X-ray diffraction (Fig. 3), indicating that two hydroxyls (C-8 and C-11) were on the opposite side. Therefore, two hydroxyls (C-8 and C-11) of **4** placed the co-facial orientation. The ECD spectrum and chiral separation chromatogram (Fig. S40) of (±)-**4** clearly demonstrated that it was a racemate. Compound (±)-**4** was successfully separated into (+)-**4** and (−)-**4** enantiomers whose absolute configurations were determined as 8*R*,11*R* and 8*S*,11*S*, respectively (Fig. 4).

Otteacumiene K (**5**), colorless needle crystals, was deduced to have a molecular formula of C_19_H_18_O_3_ according to the [M − H]^−^ ion at *m/z* 293.1178 (calcd. for C_19_H_17_O_3_, 293.1178) in negative HRESIMS, suggesting 11 degrees of unsaturation. By comparing its 1D NMR data (Tables 1 and 2) with strained cyclic diarylheptanoids tedarene B [11], the differences between **5** and tedarene B were that there were hydroxyl groups attached to C-1 (*δ*_C_ 153.7) on the benzene ring and to C-12 (*δ*_C_ 79.9) on the heptane chain, as proven by the key ^1^H-^1^H COSY correlations of H_2_-7 (*δ*_H_ 2.86, m, H-7a; 2.72, m, H-7b) /H_2_-8 (*δ*_H_ 2.22, m, H-8a; 1.98, m, H-8b) /H-9 (*δ*_H_ 3.35, m) /H-10 (*δ*_H_ 4.06, dd, *J* = 17.1, 7.1 Hz) /H-11 (*δ*_H_ 5.36, dd, *J* = 17.1, 7.6 Hz) /H-12 (*δ*_H_ 3.81, m) /H_2_-13 (*δ*_H_ 2.49, t, *J* = 12.4 Hz, H-13a; 3.10, dd, *J* = 12.4, 5.0 Hz, H-13b) and HMBC correlations from H-7 to C-3, C-4, and C-5; from H_2_-13 to C-3′, C-4′, and C-5′ (Fig. 2). The downfield aromatic quaternary carbon signals at C-2 (*δ*_C_ 125.3) and C-2′ (*δ*_C_ 120.8) suggested that **5** could be a biaryl-type cyclic diarylheptanoid derivative (Table 2), evidenced by key HMBC correlations from H-3′ (*δ*_H_ 6.38, d, *J* = 2.0 Hz) to C-2. Combining degrees of unsaturation and analysis of NMR (Tables 1 and 2), it was inferred that **5** possessed one additional cyclic system, proved by key HMBC correlations (Fig. 2) from H-9 to C-3. As a consequence, the planar structure of **5** was unambiguously determined. The result of single-crystal X-ray diffraction of **5** (Fig. 3) confirmed the relative configuration and also indicated that (±)-**5** was a racemate. However, due to the limited amount of trace samples obtained, it was unfeasible to obtain optically pure enantiomers of (±)-**5**. Therefore, the absolute configurations of (±)-**5** could not be determined.

Diarylheptanoids featuring a disubstituted heptane chain, isolated from *O.* *acuminata*, were frequently obtained as racemic mixtures. The pronounced conformational flexibility of these molecules posed challenges in definitively assigning their relative configurations using NOESY experiments. To address this, these enantiomers were subjected to chiral separation to obtain a pair of optically pure enantiomers. Subsequently, the absolute configurations of the enantiomers were determined through comparative analysis of experimental and calculated ECD spectra. Otteacumienes G–K (**1**–**5**) were identified as enantiomeric isomers. Based on structural analysis, we suppose a potential mechanism for the formation of these enantiomers: the double bond ∆(7,8) on the heptane chain of **1** and **2** underwent oxidative cyclization to form a ternary oxygen ring, followed by a ring-opening reaction that results in the generation of enantiomers.

Otteacumiene L (**6**) was isolated as a white powder and its molecular formula was determined as C_25_H_28_O_8_ by the HRESIMS at *m/z* 479.1682 [M + Na]^+^ (calculated for 479.1682), suggesting 12 degrees of unsaturation. The ^1^H and ^13^C NMR (Table 3) showed the presence of a *β*-glucopyranosyl part (*δ*_H_ 5.02, d, *J* = 7.9 Hz, H-1″) [12]. Analysis of the HRESIMS and 1D NMR data (Table 3) of compound **6** indicated that the aglycone of **6** shared identical planar structure with otteacumiene A, a known compound previously characterized by our group [8], which was confirmed by the key ^1^H-^1^H COSY correlations of H_2_-7 (*δ*_H_ 2.37, m)/H-8 (*δ*_H_ 4.24, t, *J* = 9.8 Hz) /H-9 (*δ*_H_ 5.40, t, *J* = 11.5 Hz) /H-10 (*δ*_H_ 5.91, t, *J* = 11.5 Hz) /H-11 (*δ*_H_ 5.32, dd, *J* = 15.3, 11.5 Hz) /H-12 (*δ*_H_ 6.07, dt, *J* = 15.3, 4.7 Hz) /H_2_-13 (*δ*_H_ 3.52, overlapped), accompanied by HMBC correlations from H-7 to C-3 (*δ*_C_ 118.7), C-4 (*δ*_C_ 137.7), and C-5 (*δ*_C_ 122.9); from H-13 to C-3′ (*δ*_C_ 132.5), C-4′ (*δ*_C_ 139.2), and C-5′ (*δ*_C_ 134.8) (Fig. 2). Besides, key HMBC correlations from H-1″ to C-1, combined with the chemical shift changes at C-1 (*δ*_C_ 145.7), C-2 (*δ*_C_ 154.2), C-4 (*δ*_C_ 137.7), and C-6 (*δ*_C_ 118.2) proved that the glycosyl group was attached to C-1 [13]. Consequently, the planar structure of **6** was determined.

**Table 3:** ^1^H and ^13^C NMR data of **6** in CD_3_OD

No.	*δ*_H_	*δ*_C_	No	*δ*_H_	*δ*_C_
1		145.7, s	1′		157.6, s
2		154.2, s	2′	7.25, dd (8.3, 2.5)	127.1, d
3	5.44, d (1.9)	118.7, d	3′	7.30, dd (8.3, 2.5)	132.5, d
4		137.7, s	4′		139.2, s
5	6.68, dd (8.2, 1.9)	122.9, d	5′	7.35, dd (8.3, 2.5)	134.8, d
6	7.05, d (8.2)	118.2, d	6′	7.06, dd (8.3, 2.5)	124.7, d
7	2.37, m	46.0, t	1′′	5.02, d (7.9)	103.3, d
8	4.24, t (9.8)	75.0, d	2′′	3.59, m	75.3, d
9	5.40, t (11.5)	135.6, d	3′′	3.52, m	78.2, d
10	5.91, t (11.5)	127.1, d	4′′	3.45, m	71.7, d
11	5.32, dd (15.3, 11.5)	128.4, d	5′′	3.44, m	78.5, d
12	6.07, dt (15.3, 4.7)	137.1, d	6′′	3.89, d, (12.2) 3.74, dd, (12.2, 4.6)	62.8, t
13	3.52, overlapped	38.5, t			

*δ* in ppm, *J* in Hz, and obtained at 600/150 MHz

**Scheme 1:**
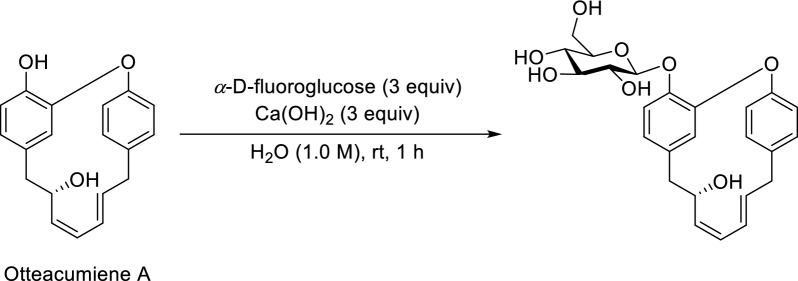
Glycosylation reaction of otteacumiene A

In order to determine the absolute configuration of **6**, it was synthesized by glycosylation. Using Ca(OH)_2_ as a base, otteacumiene A was chosen as a substrate to react with *α*-D-fluoroglucose in aqueous solvent at room temperature for 1 h (Scheme 1) [14]. Then, *O*-*β*-glc-otteacumiene A was purified by semi-preparative HPLC (MeOH/H_2_O, 50:50). Comparative analysis of the ^1^H/^13^C NMR and HRESIMS data, combined with superimposed ECD spectra, revealed that the aglycone of compound **6** had the same absolute configuration as otteacumiene A [8]. Therefore, this compound was (8*S*)-otteacumiene A 1-*O*-*β*-D-glucopyranoside, named otteacumiene L.

Otteacumiene M (**7**) was isolated as yellow oil and its molecular formula was determined as C_22_H_22_O_7_ by the HRESIMS at *m/z* 421.1261 [M + Na]^+^ (calc. for 421.1263), suggesting 12 degrees of unsaturation. Compound **7** was a new natural product. Its ^1^H NMR spectrum was completely identical to that of synthetic (*E*)-*α*-(3,4-methylenedioxybenzylidene)-*β*-(3,4,5-trimethoxybenzyl)-*γ*-butyrolactone [15], which was confirmed by the key COSY correlations of H_2_-9′/H-8′/H_2_-7′, associated with HMBC correlations from H-7 (*δ*_H_ 7.52, s) to C-2 (*δ*_C_ 108.5), C-6 (*δ*_C_ 126.8), C-8 (*δ*_C_ 126.0), and C-9 (*δ*_C_ 172.8); from H_2_-7′ (*δ*_H_ 3.01, dd, *J* = 14.9, 4.8 Hz, H-7′a; 2.63, dd, *J* = 14.9, 9.9 Hz, H-7′b) to C-8′ (*δ*_C_ 40.3), C-9′ (*δ*_C_ 70.2), C-8 (*δ*_C_ 126.0), C-1′ (*δ*_C_ 133.9), and C-2′/C-6′ (*δ*_C_ 106.1); and from -OCH_2_O- (*δ*_H_ 6.03, s) to C-3 (*δ*_C_ 149.5) and C-4 (*δ*_C_ 148.6) (Fig. 2). However, the absolute configuration of the synthetic product mentioned above was not determined. Subsequently, it was found that the known jatrophan (8′*S*, + 87) and isosuchilactone (8′*R,* − 83.3) were a pair of enantiomers [16]. By comparing their NMR data (Table 4), it was found that compound **7** was a methoxylated derivative of these two compounds at C-5′ (*δ*_C_ 153.6). These compounds had only one chiral center (C-8′). The absolute configuration of **7** was speculated to be 8′*S* by comparison of its rotation ([*α*]_D_^20^ + 27.7 (*c* 0.141, MeOH) with jatrophan (8′*S*, + 87) and isosuchilactone (8′*R,* − 83.3) [16].

**Table 4:** ^1^H and ^13^C NMR data of **7** in CDCl_3_

No.	*δ*_H_	*δ*_C_
1		128.4, s
2	7.07, d (1.6)	108.5, d
3		149.5, s
4		148.6, s
5	6.87, d (8.1)	109.0, d
6	7.05, dd (8.1, 1.6)	126.8, d
7	7.52, s	137.6, d
8		126.0, s
9		172.8, s
1′		133.9, s
2′	6.41, s	106.1, d
3′		153.6, s
4′		137.2, s
5′		153.6, s
6′	6.41, s	106.1, d
7′a	3.01, dd (14.9, 4.8) 2.63, dd (14.9, 9.9)	38.9, t
7'b		
8′	3.79, m	40.3, d
9′	4.31, m	70.2, t
-OCH_2_O-	6.03, s	102.0, t
3′-OMe	3.86, s	56.3, q
4′-OMe	3.81, s	61.1, q
5′-OMe	3.86, s	56.3, q

*δ* in parts per million, *J* in Hz, and obtained at 600/150 MHz

The inhibitory activities of compounds **1**, **2**, **4**, **6**, and** 7** against both *α*-glucosidase and PTP1B were evaluated. As summarized in Table 5, these compounds exhibited varying degrees of inhibitory activity against *α*-glucosidase at a concentration of 50 *μ*M. However, none of the tested compounds displayed detectable PTP1B inhibitory effects. An analysis of the structures and *α*-glucosidase inhibitory activities was conducted by comparing the diarylheptanoids in this study with those previously reported by our group [8]. The results of this analysis revealed that the presence and position of hydroxyl groups on the heptane chain had an impact on their *α*-glucosidase inhibitory activities. This structural-activity relationship warrants further investigation to elucidate the precise molecular mechanisms underlying the observed bioactivity.

**Table 5:** Inhibitory effects of **1**, **2**, **4**, **6**, and **7** against *α*-glucosidase^a^ and PTP1B^a^

Compounds	*α*-Glucosidase^a^	Compounds	PTP1B^a^
	Inhibition ratio (%)^b^		Inhibition ratio (%)^b^
**1**	38.97 ± 2.12	**1**	4.30 ± 1.90
(+)-**2**	25.50 ± 2.34	(+)-**2**	2.97 ± 1.32
(−)-**2**	32.32 ± 2.10	(−)-**2**	3.79 ± 1.18
**4**	20.31 ± 2.73	**4**	7.60 ± 1.11
**6**	4.71 ± 1.12	**6**	2.89 ± 1.23
**7**	7.79 ± 0.93	**7**	8.27 ± 1.82
Acarbose^c^	13.52 ± 0.46	Suramin^c^	61.65 ± 1.57

^a^Data expressed as means ± SD (n = 3)

^b^At a concentration of 50 *μ*M

^c^Positive control

## Conclusions

Five pairs of enantiomeric diarylheptanoids (**1**–**5**), one undescribed diarylheptanoid glycoside (**6**), and one new lignan (**7**) were isolated from *O.* *acuminata* var. *acuminata*. Among them, compounds **1**–**4** were individually subjected to chiral separation, yielding optically pure enantiomers for each. The absolute configurations of these enantiomers were determined using X-ray crystallography and by comparing the calculated and experimental ECD curves. Notably, compound **6** was the first diarylheptanoid glycoside isolated from this plant, and its absolute configuration was confirmed by semi-synthesis. This study enriched the fundamentals of chemical substances of the title plant. Furthermore, at a concentration of 50 *μ*M, all the tested compounds exhibited varying degrees of inhibitory potency against *α*-glucosidase, which provides a clue for the development of hypoglycemic drugs.

## Experimental procedures

### General experimental procedures

Optical rotations were recorded on a JASCO P-1020 polarimeter. The UV spectra were recorded with a Shimadzu UV-2401PC spectrometer. HRESIMS analysis was performed with Agilent G6230 TOF mass spectrometers. 1D and 2D NMR spectra were obtained with a Bruker DRX-600 spectrometer using TMS as an internal standard. The chemical shifts (*δ*) were expressed in ppm with reference to the solvent signals. Semi-preparative HPLC was performed on a Waters 1525 HPLC with a ZORBAX SB-C18 (9.4 × 250 mm) column. Silica gel (100–200 and 200–300 mesh, Qingdao Marine Chemical Co., Ltd., People’s Republic of China). Fractions were monitored by TLC (GF 254, Qingdao Marine Chemical Co., Ltd.), and spots were visualized by heating silica gel plates immersed in 10% H_2_SO_4_ in ethanol. All industrial-grade solvents were re-distilled before use. Otteacumiene A is an isolate obtained by our group in the early stage. *α*-Glucosidase, 4-nitrophenyl-*α*-D-glucopyranoside (PNPG), and acarbose were purchased from Sigma. PTP1B was purchased from Sino Biological. Disodium 4-nitrophenylphosphate (PNPP) was purchased from MeilunBio.

### Plant material

The plants of *O.* *acuminata* var. *acuminata* were collected in Dali Prefecture (Yunnan, China) on October 2019, it was identified by Prof. Yun-Heng Ji in Kunming Institute of Botany, Chinese Academy of Sciences. A voucher specimen (KIB L-20191001) was deposited in Kunming Institute of Botany.

### Extraction and isolation

The dried *O.* *acuminata* var. *acuminata* (180.0 kg) were soaked and extracted with methanol, and the complete evaporation of the solvent gave crude extract (13.5 kg). The crude extract was eluted with methanol: water (1:1) through macroporous resin to afford a fraction (505.9 g). This fraction was suspended in distilled water and partitioned three times with EtOAc to obtain a fraction (179.0 g). The EtOAc fraction was subjected to MCI-gel column with a gradient system (MeOH-H_2_O from 3:7 to 10:0) to produce eight fractions (Frs. A–H). Frs. A–H were extracted and purified by silica gel, RP-C18, Sephadex gel, and preparative or semi-preparative HPLC-gel chromatographic methods to obtain the nine isolates.

Fr. C (10.2 g) was chromatographed on a silica gel column with petroleum ether (PE)/acetone (AC) (200:1–0:1, *v*/*v*), to yield seven fractions (Frs. C1–C7). Fr. C3 (436.3 mg) was divided into seven sub-fractions (Frs. C3-1–C3-7) by Sephadex gel column with acetone. Fr. C3-2 (91.1 mg) was further separated by semi-preparative HPLC-gel (CH_3_CN/H_2_O, 70:30, *v*/*v*) to obtain **7** (4.0 mg).

Fr. E (68.4 g) was divided into nine sub-fractions (Frs. E1–E9) by a silica gel column with PE/ AC (50:1–0:1, *v*/*v*). Fr. E8 (10.3 g) was further chromatographed over a RP-C18 column, eluted with MeOH/H_2_O (3:7 to 100:0), to yield eight fractions (Frs. E8-1–E8-8). Fr. E8-1 (106.5 mg) was subjected to Sephadex LH-20 (MeOH), followed by semi-preparative HPLC (CH_3_CN/H_2_O, 38:62) to afford **5** (1.2 mg). Fr. E8-4 (356.6 mg) was purified by preparative HPLC (MeOH/H_2_O, 45:55) and semi-preparative HPLC (CH_3_CN/H_2_O, 35:65, *v*/*v*) to get **3** (1.0 mg) and **4** (5.0 mg). Fr. E8-5 (277.6 mg) was purified by preparative HPLC (MeOH/H_2_O, 45:55, *v*/*v*) and semi-preparative HPLC (CH_3_CN/H_2_O, 35:65) to obtain **1** (2.4 mg) and **2** (2.1 mg). Fr. E9 (5.5 g) was subjected on a RP-18 column chromatography (MeOH/H_2_O, 3:7 to 10:0) to afford six fractions (Frs. E9-1–E9-6). Fr. E9-4 (337.2 mg) was divided into four fractions (Frs. E9-4-1–E9-4-4) by Sephadex gel column with methanol. Fr. E9-4-4 (63.7 mg) was separated by semi-preparative HPLC (MeOH/H_2_O, 50:50) to afford **6** (3.4 mg).

Otteacumiene G (**1**): Colorless crystals; IR (*v*_max_) 3426, 2926, 1613, 1596, 1516, 1502, 1436, 1210, 1106, 836 cm^−1^; UV (MeOH) *λ*_max_ (log *ε*) 278 (2.25) nm; HRESIMS *m/z* 311.1287 [M − H]^−^ (calculated for C_19_H_19_O_4,_ 311.1289); ^1^H and ^13^C NMR spectroscopic data see Tables 1 and 2. (+)-**1**: [*α*]_D_^20^ + 40.0 (*c* 0.037, MeOH); ECD (MeOH) *λ*_max_ (*Δε*) 285 (− 0.94), 269 (1.04), 240 (5.35), 223 (− 0.20), 213 (3.24), 199 (8.30) nm; (−)-**1**: [*α*]_D_^20^ − 35.0 (*c* 0.040, MeOH); ECD (MeOH) *λ*_max_ (*Δε*) 284 (1.1), 270 (− 0.5), 241 (− 4.0), 223 (1.2), 214 (− 1.5), 199 (− 5.9) nm.

Otteacumiene H (**2**): Colorless crystals; IR (*v*_max_) 3414, 2926, 1596, 1503, 1436, 1210, 1108, 848 cm^−1^; UV (MeOH) *λ*_max_ (log *ε*) 276 (2.22) nm; HRESIMS *m/z* 311.1287 [M − H]^−^ (calculated for C_19_H_19_O_4,_ 311.1289); ^1^H and ^13^C NMR spectroscopic data see Tables 1 and 2. ( +)-**2**: [*α*]_D_^25^ + 29.2 (*c* 0.048, MeOH); ECD (MeOH) *λ*_max_ (*Δε*) 285 (− 1.4), 270 (0.8), 242 (5.5), 216 (1.5), 206 (− 4.5), 197 (9.3) nm; ( −)-**2**: [*α*]_D_^25^ − 37.7 (*c* 0.052, MeOH); ECD (MeOH) *λ*_max_ (*Δε*) 284 (1.3), 270 (− 4.5), 242 (− 4.1), 216 (− 0.8), 206 (4.1), 197 (− 6.4) nm.

Otteacumiene I (**3**): Colorless crystals; IR (*v*_max_) 3426, 2922, 1629, 1604, 1412, 1384, 1217, 8337 cm^−1^; UV (MeOH) *λ*_max_ (log *ε*) 279 (2.17) nm; HRESIMS *m/z* 311.1286 [M − H]^−^ (calculated for C_19_H_19_O_4,_ 311.1289); ^1^H and ^13^C NMR spectroscopic data see Tables 1 and 2. ( +)-**3**: [*α*]_D_^20^ + 4.59 (*c* 0.048, MeOH); ECD (MeOH) *λ*_max_ (*Δε*) 283 (− 0.8), 240 (2.3), 221 (− 2.7), 198 (2.9) nm; ( −)-**3**: [*α*]_D_^20^ − 4.00 (*c* 0.050, MeOH); ECD (MeOH) *λ*_max_ (*Δε*) 282 (0.6), 239 (− 2.8), 221 (2.4), 199 (− 3.3) nm.

Otteacumiene J (**4**): White powder; IR (*v*_max_) 3431, 2929, 1518,1504, 1273, 1210, 1112, 1029, 837, 574 cm^−1^; UV (MeOH) *λ*_max_ (log *ε*) 279 (2.25) nm; HRESIMS *m/z* 311.1285 [M − H]^−^ (calculated for C_19_H_19_O_4,_ 311.1283); ^1^H and ^13^C NMR spectroscopic data see Tables 1 and 2. ( +)-**4**: [*α*]_D_^20^ + 4.48 (*c* 0.067, MeOH); ECD (MeOH) *λ*_max_ (*Δε*) 285 (0.2), 270 (− 0.4), 255 (− 0.2), 226 (2.6), 201 (− 7.8), 195 (− 4.2) nm; ( −)-**4**: [*α*]_D_^20^ − 6.08 (*c* 0.092, MeOH); ECD (MeOH) *λ*_max_ (*Δε*) 286 (– 0.3), 271 (0.3), 255 (0.2), 226 (− 2.9), 201 (8.5), 195 (4.9) nm.

Otteacumiene K (**5**)**:** Colorless needle crystal; [*α*]_D_^20^ − 3.17 (*c* 0.145, MeOH); IR (*v*_max_) 3422, 2925, 1607, 1412, 1242, 813, 562 cm^−1^; UV (MeOH) *λ*_max_ (log *ε*) 290 (2.70) nm; HRESIMS *m/z* 293.1178 [M − H]^−^ (calculated for C_19_H_17_O_3,_ 293.1178); ^1^H and ^13^C NMR spectroscopic data see Tables 1 and 2.

Otteacumiene L (**6**): White powder; [*α*]_D_^25^ + 1.1 (*c* 0.195, MeOH); ECD (MeOH) *λ*_max_ (*Δε*) 284 (3.1), 269 (− 0.1), 242 (10.6), 197 (− 27.6) nm; UV (MeOH) *λ*_max_ (log *ε*) 278 (2.22) nm; IR (*v*_max_) 3413, 2921, 1587, 1510, 1416, 1254, 1201, 1073, 868 cm^−1^; HRESIMS *m/z* 479.1682 [M + Na]^+^ (calculated for C_25_H_28_O_8_Na, 479.1682); ^1^H and ^13^C spectroscopic data see Table 3.

Otteacumiene M (**7**): Yellow oil; [*α*]_D_^20^ + 27.7 (*c* 0.141, MeOH); ECD (MeOH) *λ*_max_ (*Δε*) 338 (1.0), 287 (− 2.3), 247 (1.3), 239 (0.8), 231 (1.7), 220 (0.1), 209 (1.5), 197 (− 1.2) nm; UV (MeOH) *λ*_max_ (log *ε*) 205 (3.52), 297 (2.82), 334 (3.02) nm; IR (*v*_max_) 3435, 2940, 1749, 1640, 1591, 1502, 1450, 1342, 1241, 1128 cm^−1^; HRESIMS *m/z* 421.1261 ([M + Na]^+^ (calculated for C_22_H_22_O_7_Na, 421.1263); ^1^H and ^13^C spectroscopic data see Table 4.

Crystal data for** 1**: C_19_H_20_O_4_, *M* = 312.35, *a* = 7.3795(2) Å, *b* = 15.4127(4) Å, *c* = 14.0622(4) Å, *α* = 90°, *β* = 104.6890(10)°, *γ* = 90°, *V* = 1547.13(7) Å^3^, *T* = 150.(2) K, space group *P*121*/c*1, *Z* = 4, *μ*(Cu Kα) = 0.759 mm^−1^, 13,446 reflections measured, 2809 independent reflections (*R*_*int*_ = 0.0507). The final *R*_*1*_ values were 0.0424 (*I* > 2*σ*(*I*)). The final *wR*(*F*^2^) values were 0.1080 (*I* > 2*σ*(*I*)). The final *R*_*1*_ values were 0.0462 (all data). The final *wR*(*F*^2^) values were 0.1111 (all data). The goodness of fit on *F*^2^ was 1.040.

Crystal data for **3**: C_19_H_20_O_4_, *M* = 312.35, *a* = 9.1373(3) Å, *b* = 10.3847(3) Å, *c* = 16.6741(5) Å, *α* = 90°, *β* = 102.2910(10)°, *γ* = 90°, *V* = 1545.91(8) Å^3^, *T* = 150.(2) K, space group *P*121*/c*1, *Z* = 4, *μ*(Cu Kα) = 0.760 mm^−1^, 26,920 reflections measured, 3031 independent reflections (*R*_*int*_ = 0.0585). The final *R*_*1*_ values were 0.0376 (*I* > 2*σ*(*I*)). The final *wR*(*F*^2^) values were 0.0913 (*I* > 2*σ*(*I*)). The final *R*_*1*_ values were 0.0404 (all data). The final *wR*(*F*^2^) values were 0.0932 (all data). The goodness of fit on *F*^2^ was 1.024.

Crystal data for** 5**: Crystal data for rxz160x: C_19_H_18_O_3_, *M* = 294.33, *a* = 8.9038(4) Å, *b* = 9.4697(4) Å, *c* = 17.1220(7) Å, *α* = 90°, *β* = 90°, *γ* = 90°, *V* = 1443.66(11) Å^3^, *T* = 150. (2) K, space group *Pna*21, *Z* = 4, *μ*(Cu Kα) = 0.729 mm^−1^, 19,243 reflections measured, 2586 independent reflections (*R*_*int*_ = 0.1368). The final *R*_*1*_ values were 0.0338 (*I* > 2*σ*(*I*)). The final *wR*(*F*^2^) values were 0.0804 (*I* > 2*σ*(*I*)). The final *R*_*1*_ values were 0.0418 (all data). The final *wR*(*F*^2^) values were 0.0828 (all data). The goodness of fit on *F*^2^ was 1.043. Flack parameter = 0.55(13).

### *α*‑Glucosidase and PTB1B inhibitory activities detection

*α*-Glucosidase inhibitory assay: enzyme solution (0.025 U/mL), buffer, and substrate (1 mM) were sequentially added to the enzyme labeled plates along with the samples and three-well replicates were established for each sample. A blank control without drug and a positive control of acarbose were also set up. The plate was incubated at 37 °C for 50 min and the OD value at 405 nm was measured by enzyme marker to calculate the inhibition rate of *α*-glucosidase activity.

PTB1B inhibitory assay: the procedure was similar to *α*-glucosidase inhibitory assay. Incubate at 37 ℃ for 30 min, then add Na_2_CO_3_ termination solution. Finally, the OD value was determined by an enzyme meter with a detection wavelength of 405 nm (positive control was suramin).

The inhibition percentage: inhibition rate (%) = (1−S/E) × 100% (S is the OD of the sample, E is the OD of the control).

## Supplementary Information

Supplementary material 1.

## Data Availability

The datasets used or analyzed during the current study are available from the corresponding author on reasonable request.
