# Supplementary material for: Enantiomeric diarylheptanoids from Ottelia acuminata var. acuminata and their α-glucosidase inhibitory activity
Source: Nat Prod Bioprospect. 2025 May 16;15(1):33. doi: 10.1007/s13659-025-00515-w (PMC12084200; doi:10.1007/s13659-025-00515-w)
Supplement: Supplementary file 1 — Supplementary material 1. [file 13659_2025_515_MOESM1_ESM.docx]

**SUPPLEMENTARY INFORMATION**

Enantiomeric diarylheptanoids from *Ottelia acuminata* var. *acuminata* and their *α*-glucosidase inhibitory activity

Jia-Ru Zhou^1, 2, †^, Xin-Yue Hu^1, 2, †^, Hong-Xing Liu^1^, Yu Zhou^1, 2^, Fei-Fei Xiong^1, 2^, Jian-Jun Zhao^1^, Xing-Ren Li^1,^ ^[[1]](#footnote-1)^* and Gang Xu^1,^ ^[[2]](#footnote-2)^*

^1^State Key Laboratory of Phytochemistry and Natural Medicines, and Yunnan Key Laboratory of Natural Medicinal Chemistry, Kunming Institute of Botany, Chinese Academy of Sciences, Kunming, 650201, China;

^2^University of Chinese Academy of Sciences, Beijing, 100049, China.

**Content**

[Figure S1. Individuals of racemic mixtures (±)-1 isolated by chiral resolution 6](#_Toc195361861)

[Figure S2. ^1^H NMR spectrum of 1 6](#_Toc195361862)

[Figure S3. ^13^C NMR spectrum of 1 7](#_Toc195361863)

[Figure S4. HMQC spectrum of 1 7](#_Toc195361864)

[Figure S5. HMBC spectrum of 1 8](#_Toc195361865)

[Figure S6. ^1^H-^1^H COSY spectrum of 1 8](#_Toc195361866)

[Figure S7. ROESY spectrum of 1 9](#_Toc195361867)

[Figure S8. HRESIMS spectrum of 1 10](#_Toc195361868)

[Figure S9. IR spectrum of 1 11](#_Toc195361869)

[Figure S10. ECD spectrum of (+)-1 11](#_Toc195361870)

[Figure S11. UV spectrum of (+)-1 12](#_Toc195361871)

[Figure S12. ECD spectrum of (−)-1 12](#_Toc195361872)

[Figure S13. UV spectrum of (−)-1 13](#_Toc195361873)

[Figure S14. Individuals of racemic mixtures (±)-2 isolated by chiral resolution 13](#_Toc195361874)

[Figure S15. ^1^H NMR spectrum of 2 14](#_Toc195361875)

[Figure S16. ^13^C NMR spectrum of 2 14](#_Toc195361876)

[Figure S17. HMQC spectrum of 2 15](#_Toc195361877)

[Figure S18. HMBC spectrum of 2 15](#_Toc195361878)

[Figure S19. ^1^H-^1^H COSY spectrum of 2 16](#_Toc195361879)

[Figure S20. ROESY spectrum of 2 16](#_Toc195361880)

[Figure S21. HRESIMS spectrum of 2 17](#_Toc195361881)

[Figure S22. IR spectrum of 2 18](#_Toc195361882)

[Figure S23. ECD spectrum of (+)-2 18](#_Toc195361883)

[Figure S24. UV spectrum of (+)-2 19](#_Toc195361884)

[Figure S25. ECD spectrum of (−)-2 19](#_Toc195361885)

[Figure S26. UV spectrum of (−)-2 20](#_Toc195361886)

[Figure S27. Individuals of racemic mixtures (±)-3 isolated by chiral resolution 20](#_Toc195361887)

[Figure S28. ^1^H NMR spectrum of 3 21](#_Toc195361888)

[Figure S29. ^13^C NMR spectrum of 3 21](#_Toc195361889)

[Figure S30. HMQC spectrum of 3 22](#_Toc195361890)

[Figure S31. HMBC spectrum of 3 22](#_Toc195361891)

[Figure S32. ^1^H-^1^H COSY spectrum of 3 23](#_Toc195361892)

[Figure S33. ROESY spectrum of 3 23](#_Toc195361893)

[Figure S34. HRESIMS spectrum of 3 24](#_Toc195361894)

[Figure S35. IR spectrum of 3 25](#_Toc195361895)

[Figure S36. ECD spectrum of (+)-3 25](#_Toc195361896)

[Figure S37. UV spectrum of (+)-3 26](#_Toc195361897)

[Figure S38. ECD spectrum of (−)-3 26](#_Toc195361898)

[Figure S39. UV spectrum of (−)-3 27](#_Toc195361899)

[Figure S40. Individuals of racemic mixtures (±)-4 isolated by chiral resolution 27](#_Toc195361900)

[Figure S41. ^1^H NMR spectrum of 4 28](#_Toc195361901)

[Figure S42. ^13^C NMR spectrum of 4 28](#_Toc195361902)

[Figure S43. HMQC spectrum of 4 29](#_Toc195361903)

[Figure S44. HMBC spectrum of 4 29](#_Toc195361904)

[Figure S45. ^1^H-^1^H COSY spectrum of 4 30](#_Toc195361905)

[Figure S46. ROESY spectrum of 4 30](#_Toc195361906)

[Figure S47. HRESIMS spectrum of 4 31](#_Toc195361907)

[Figure S48. IR spectrum of 4 32](#_Toc195361908)

[Figure S49. ECD spectrum of (+)-4 32](#_Toc195361909)

[Figure S50. UV spectrum of (+)-4 33](#_Toc195361910)

[Figure S51. ECD spectrum of (−)-4 33](#_Toc195361911)

[Figure S52. UV spectrum of (−)-4 34](#_Toc195361912)

[Figure S53. ^1^H NMR spectrum of 5 34](#_Toc195361913)

[Figure S54. ^13^C NMR spectrum of 5 35](#_Toc195361914)

[Figure S55. HMQC spectrum of 5 35](#_Toc195361915)

[Figure S56. HMBC spectrum of 5 36](#_Toc195361916)

[Figure S57. ^1^H-^1^H COSY spectrum of 5 36](#_Toc195361917)

[Figure S58. ROESY spectrum of 5 37](#_Toc195361918)

[Figure S59. HRESIMS spectrum of 5 38](#_Toc195361919)

[Figure S60. IR spectrum of 5 39](#_Toc195361920)

[Figure S61. ECD spectrum of 5 39](#_Toc195361921)

[Figure S62. UV spectrum of 5 40](#_Toc195361922)

[Figure S63. ^1^H NMR spectrum of 6 40](#_Toc195361923)

[Figure S64. ^13^C NMR spectrum of 6 41](#_Toc195361924)

[Figure S65. HMQC spectrum of 6 41](#_Toc195361925)

[Figure S66. HMBC spectrum of 6 42](#_Toc195361926)

[Figure S67. ^1^H-^1^H COSY spectrum of 6 42](#_Toc195361927)

[Figure S68. ROESY spectrum of 6 43](#_Toc195361928)

[Figure S69. HRESIMS spectrum of 6 44](#_Toc195361929)

[Figure S70. IR spectrum of 6 45](#_Toc195361930)

[Figure S71. ECD spectrum of 6 45](#_Toc195361931)

[Figure S72. UV spectrum of 6 46](#_Toc195361932)

[Figure S73. ^1^H NMR spectrum of synthetic 6 46](#_Toc195361933)

[Figure S74. ^13^C NMR spectrum of synthetic 6 47](#_Toc195361934)

[Figure S75. HRESIMS spectrum of synthetic 6 48](#_Toc195361935)

[Figure S76. ECD spectrum of synthetic 6 49](#_Toc195361936)

[Figure S77. UV spectrum of synthetic 6 49](#_Toc195361937)

[Figure S78. ^1^H NMR spectrum of 7 50](#_Toc195361938)

[Figure S79. ^13^C NMR spectrum of 7 50](#_Toc195361939)

[Figure S80. HMQC spectrum of 7 51](#_Toc195361940)

[Figure S81. HMBC spectrum of 7 51](#_Toc195361941)

[Figure S82. ^1^H-^1^H COSY spectrum of 7 52](#_Toc195361942)

[Figure S83. ROESY spectrum of 7 52](#_Toc195361943)

[Figure S84. HRESIMS spectrum of 7 53](#_Toc195361944)

[Figure S85. IR spectrum of 7 54](#_Toc195361945)

[Figure S86. ECD spectrum of 7 54](#_Toc195361946)

[Figure S87. UV spectrum of 7 55](#_Toc195361947)

[Figure S88. Inhibitory effects of 1, 2, 4, 6, and 7 against *α*-glucosidase 55](#_Toc195361948)

[Part 1. X-ray crystal structure of 1 55](#_Toc195361949)

[Part 2. X-ray crystal structure of 3 58](#_Toc195361950)

[Part 3. X-ray crystal structure of 5 60](#_Toc195361951)

[Part 4. Calculated ECD data of compounds 1 62](#_Toc195361952)

[Part 5. Calculated ECD data of compounds 2 67](#_Toc195361953)

[Part 6. Calculated ECD data of compounds 3 70](#_Toc195361954)

[Part 7. Calculated ECD data of compounds 4 74](#_Toc195361955)

[Part 8. General Procedure of (8*S*)-otteacumiene A-*O*-*β*-D-glucopyranoside 77](#_Toc195361956)

# Figure S1. Individuals of racemic mixtures (±)-1 isolated by chiral resolution


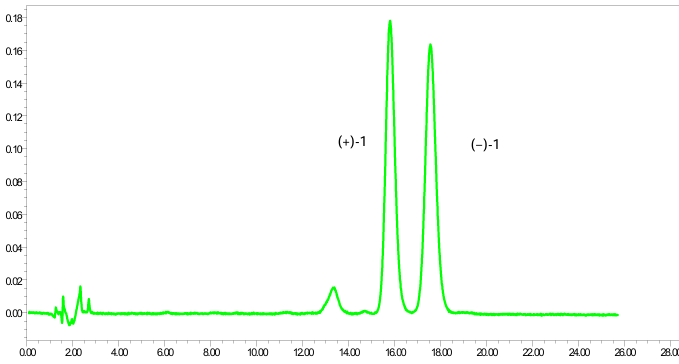


DAICEL CORPORATION semi-preparative column (CNCH_3_/H_2_O, 23:77, *v/v*)

# Figure S2. ^1^H NMR spectrum of 1

# Figure S3. ^13^C NMR spectrum of 1


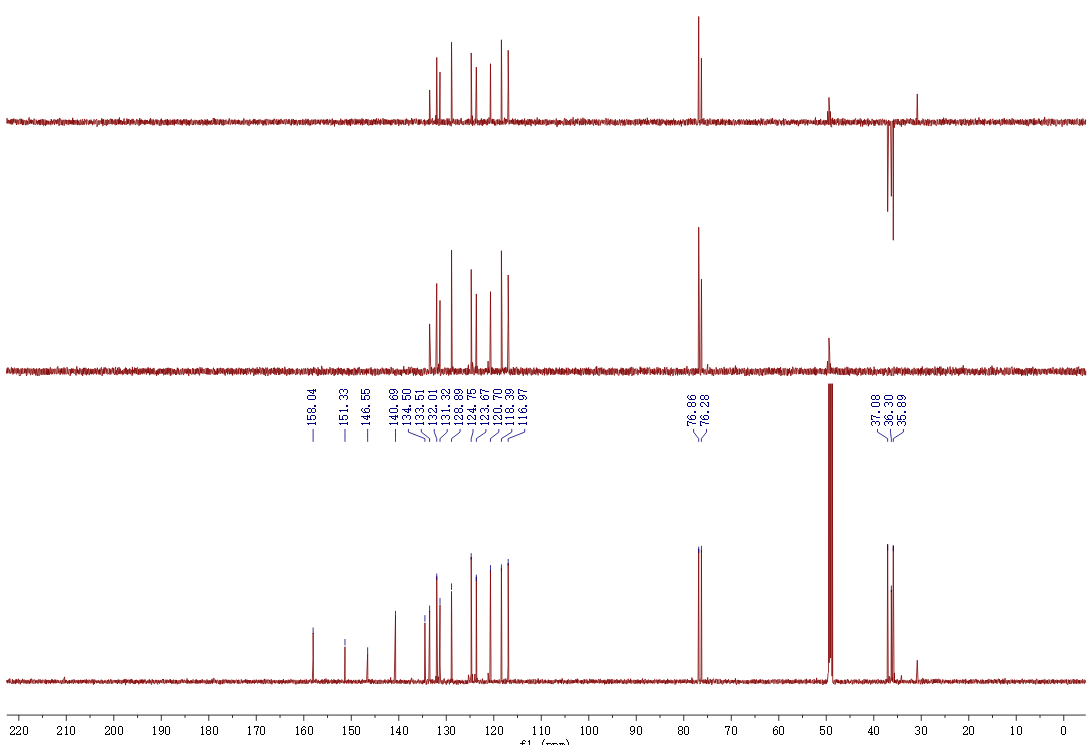


# Figure S4. HMQC spectrum of 1

# Figure S5. HMBC spectrum of 1

# Figure S6. ^1^H-^1^H COSY spectrum of 1

# Figure S7. ROESY spectrum of 1

# **Figure S8.** HRESIMS spectrum of **1**


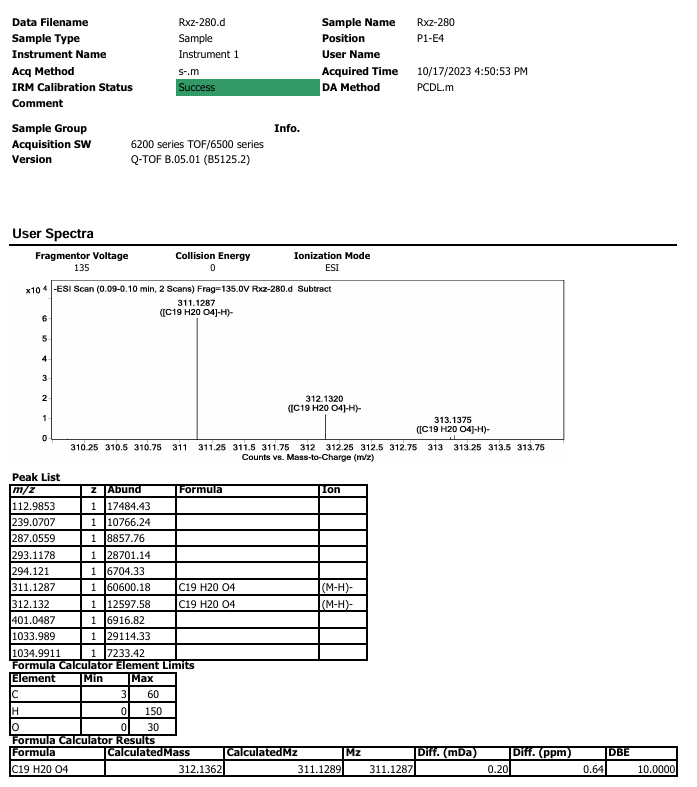


# Figure S9. IR spectrum of 1


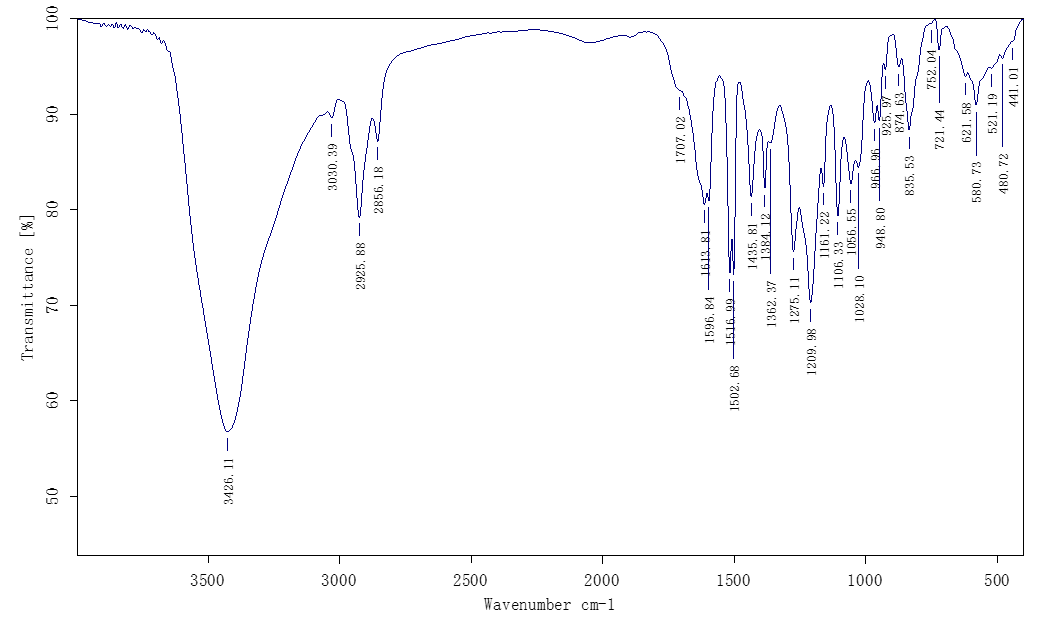


# Figure S10. ECD spectrum of (+)-1


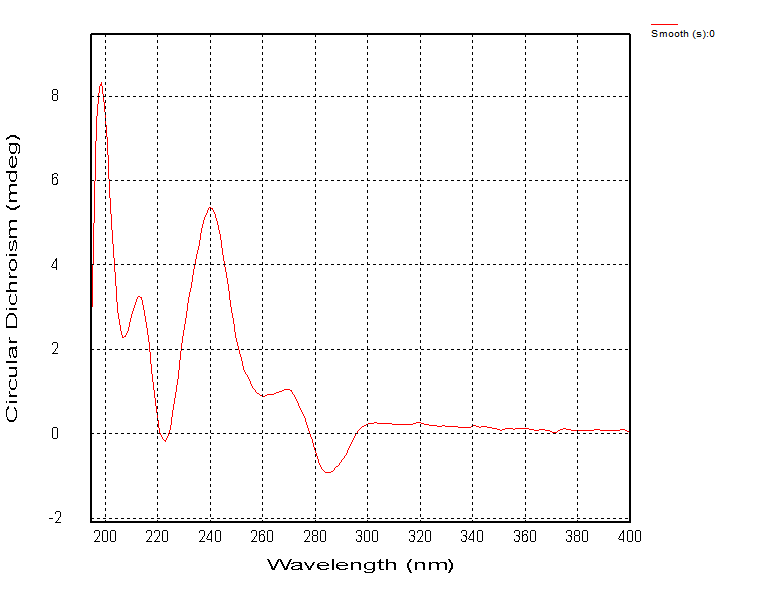


# Figure S11. UV spectrum of (+)-1


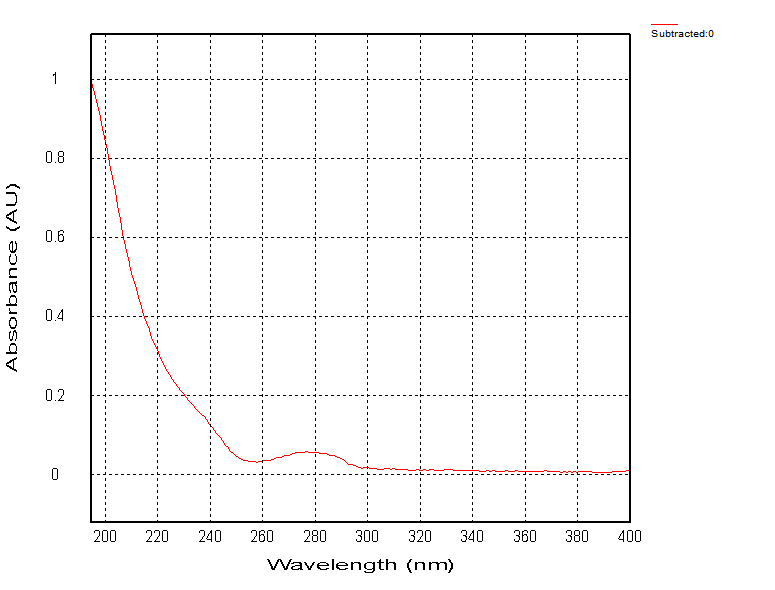


# Figure S12. ECD spectrum of (−)-1


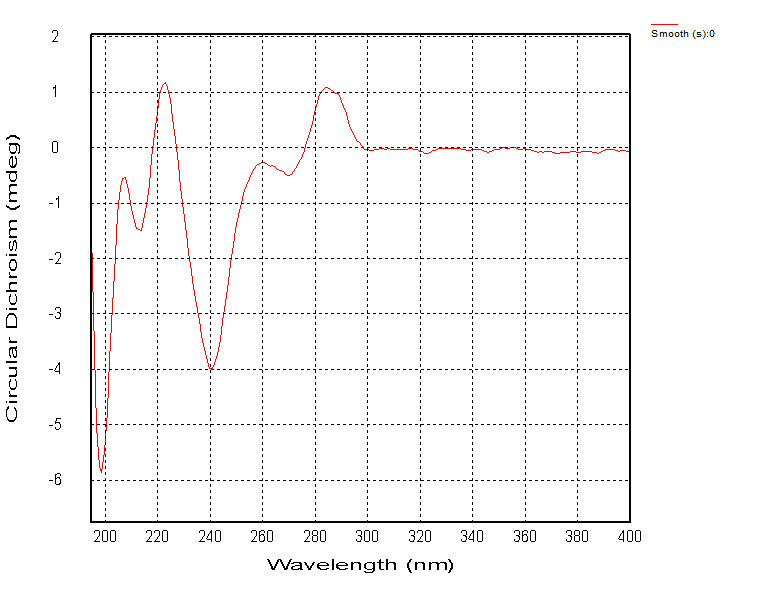


# Figure S13. UV spectrum of (−)-1


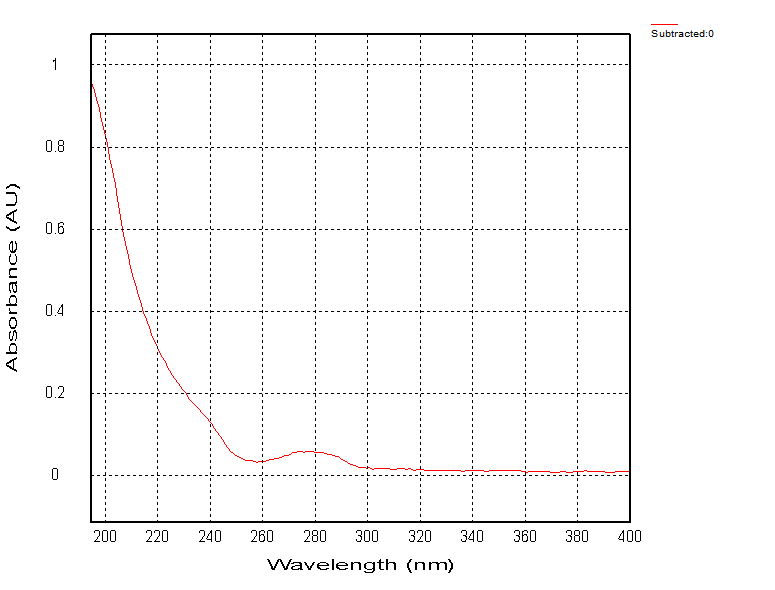


# Figure S14. Individuals of racemic mixtures (±)-2 isolated by chiral resolution


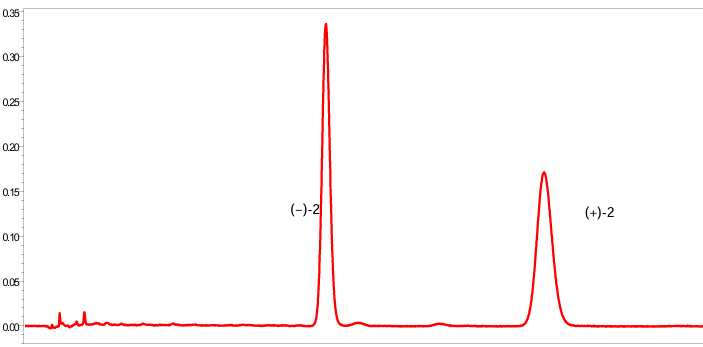


DAICEL CORPORATION semi-preparative column (CNCH_3_/H_2_O, 25:75, *v/v*)

# Figure S15. ^1^H NMR spectrum of 2

# Figure S16. ^13^C NMR spectrum of 2


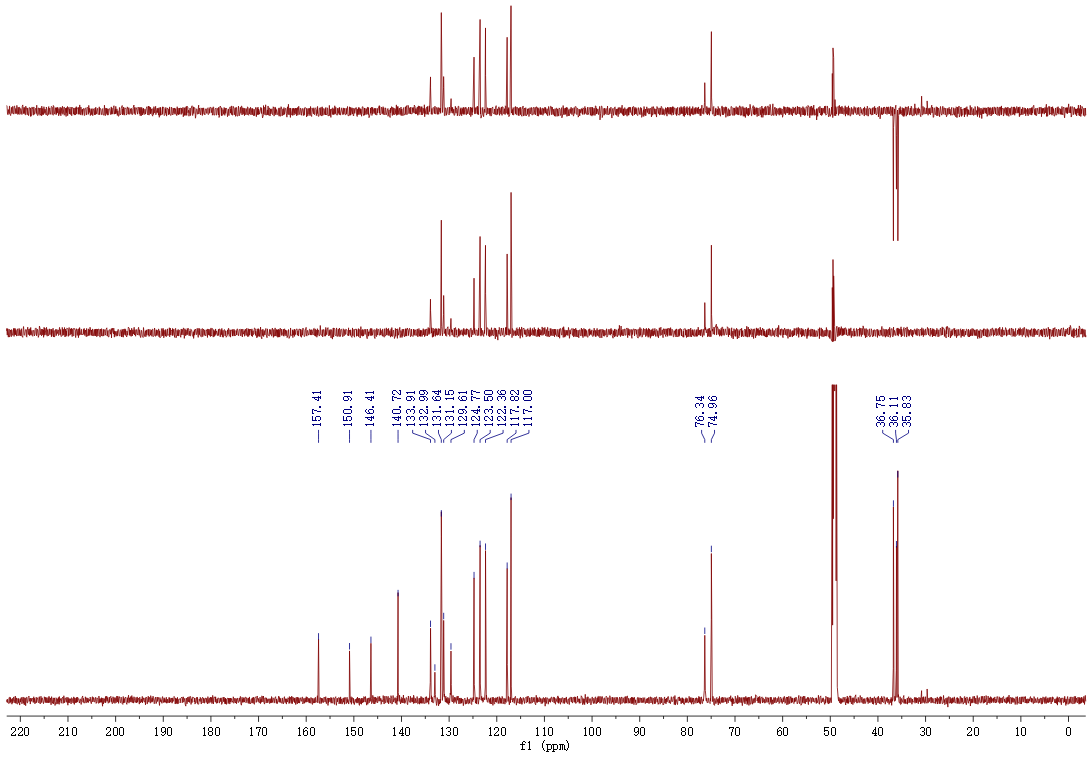


# Figure S17. HMQC spectrum of 2

# Figure S18. HMBC spectrum of 2

# Figure S19. ^1^H-^1^H COSY spectrum of 2

# Figure S20. ROESY spectrum of 2

# Figure S21. HRESIMS spectrum of 2


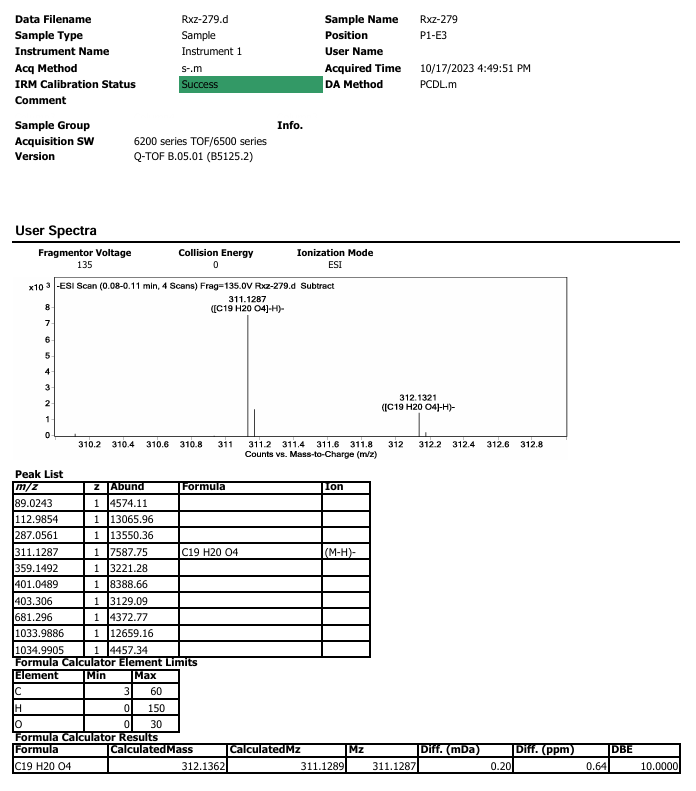


# Figure S22. IR spectrum of 2


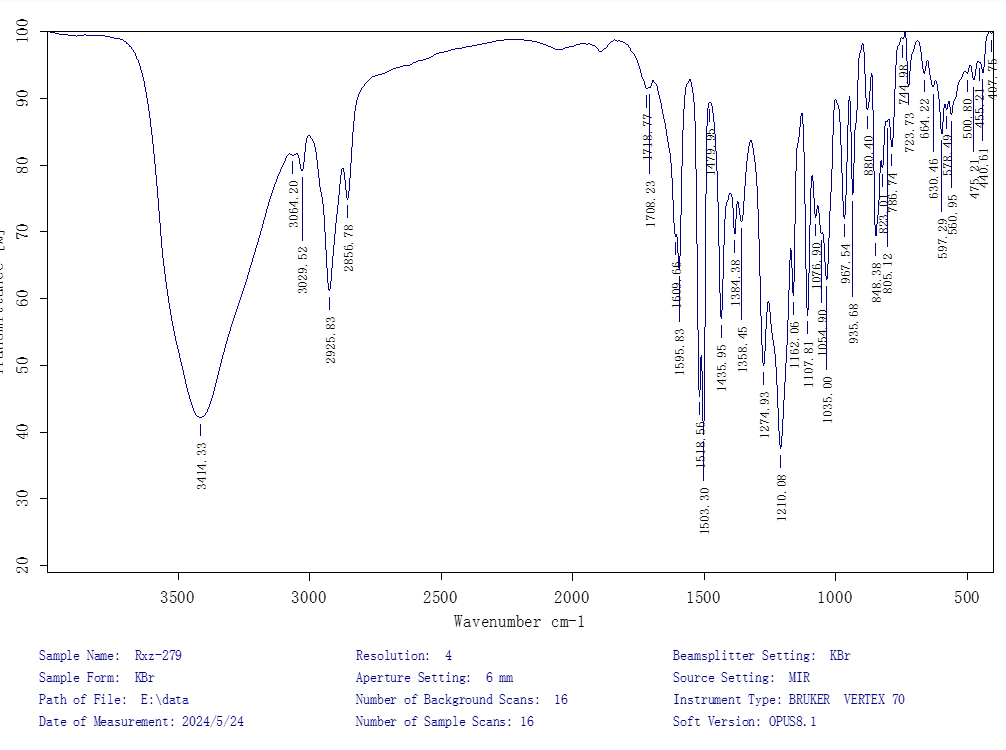


# Figure S23. ECD spectrum of (+)-2


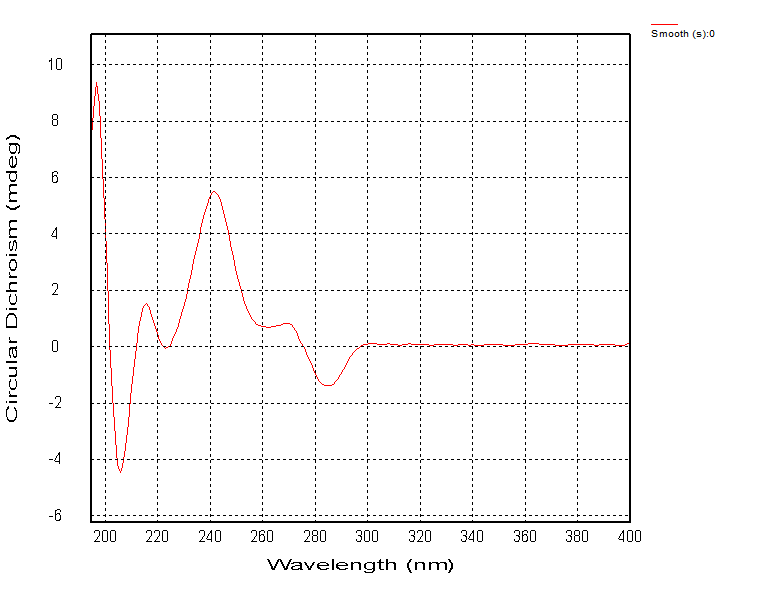


# Figure S24. UV spectrum of (+)-2


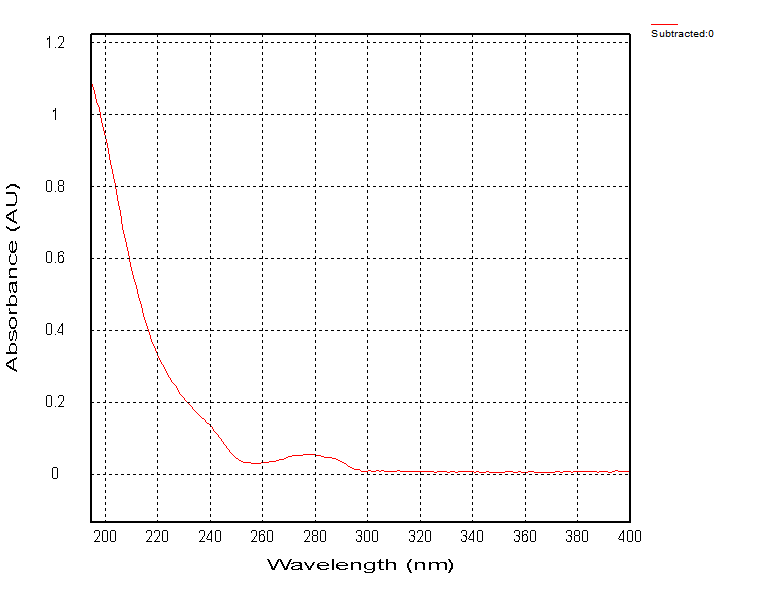


# Figure S25. ECD spectrum of (−)-2


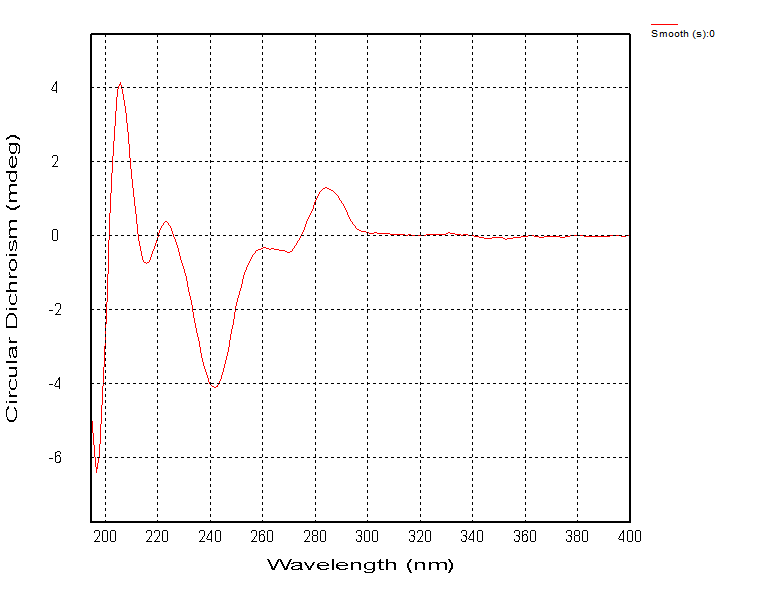


# Figure S26. UV spectrum of (−)-2


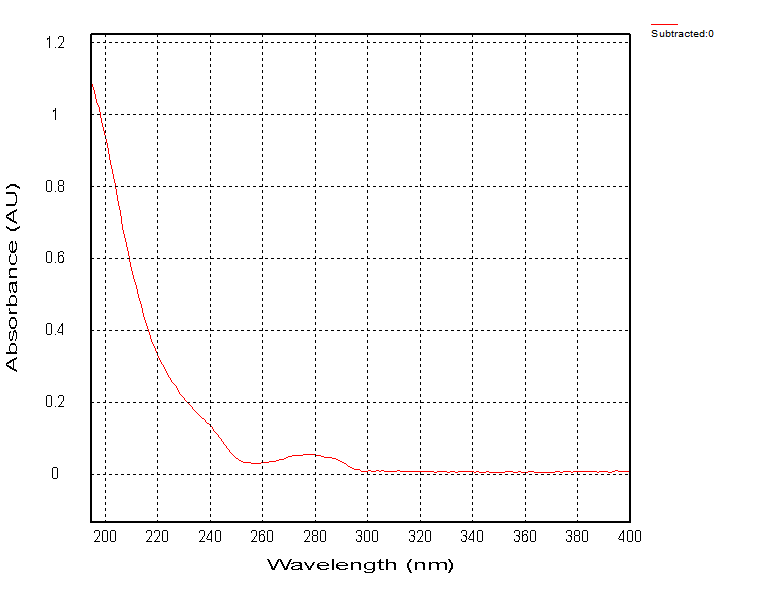


# Figure S27. Individuals of racemic mixtures (±)-3 isolated by chiral resolution


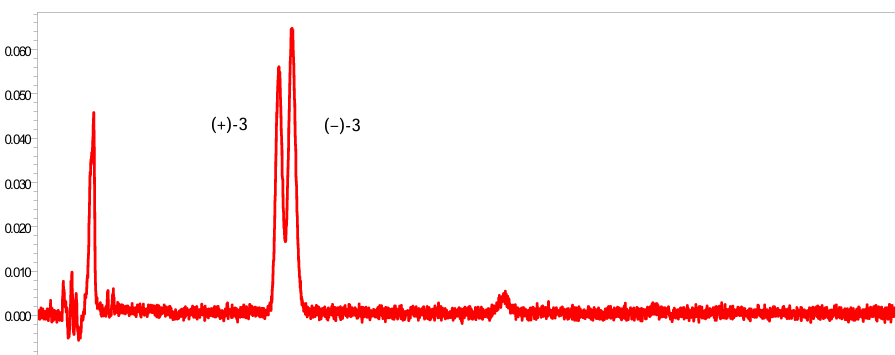


DAICEL CORPORATION semi-preparative column (CNCH_3_/H_2_O, 20:80, *v/v*)

# Figure S28. ^1^H NMR spectrum of 3

# Figure S29. ^13^C NMR spectrum of 3

# Figure S30. HMQC spectrum of 3

# Figure S31. HMBC spectrum of 3

# Figure S32. ^1^H-^1^H COSY spectrum of 3

# Figure S33. ROESY spectrum of 3


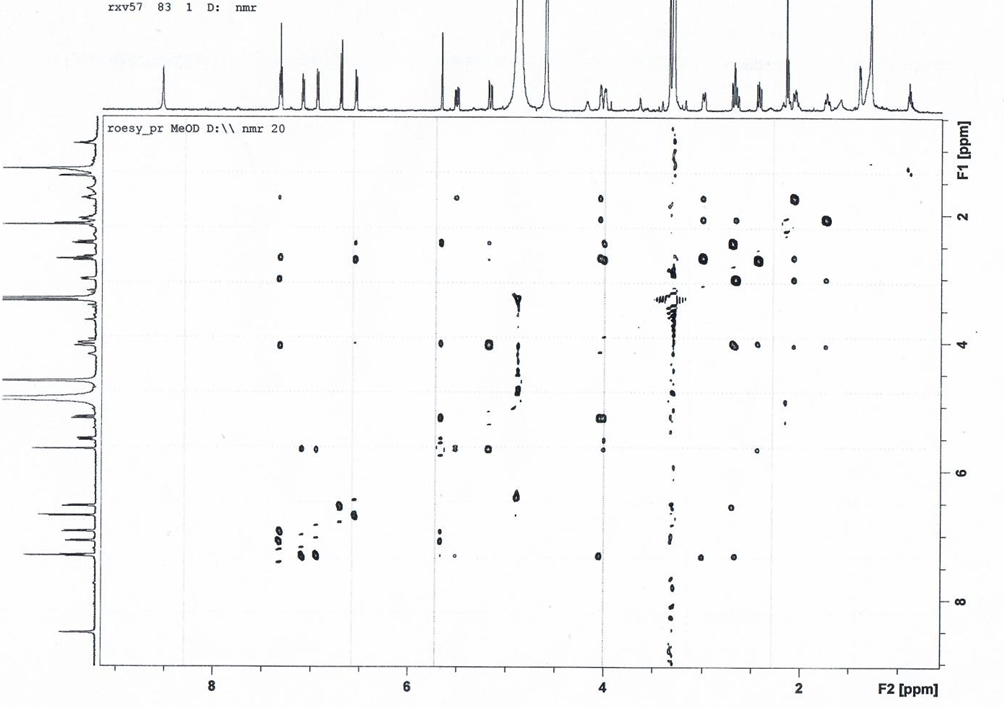


# Figure S34. HRESIMS spectrum of 3


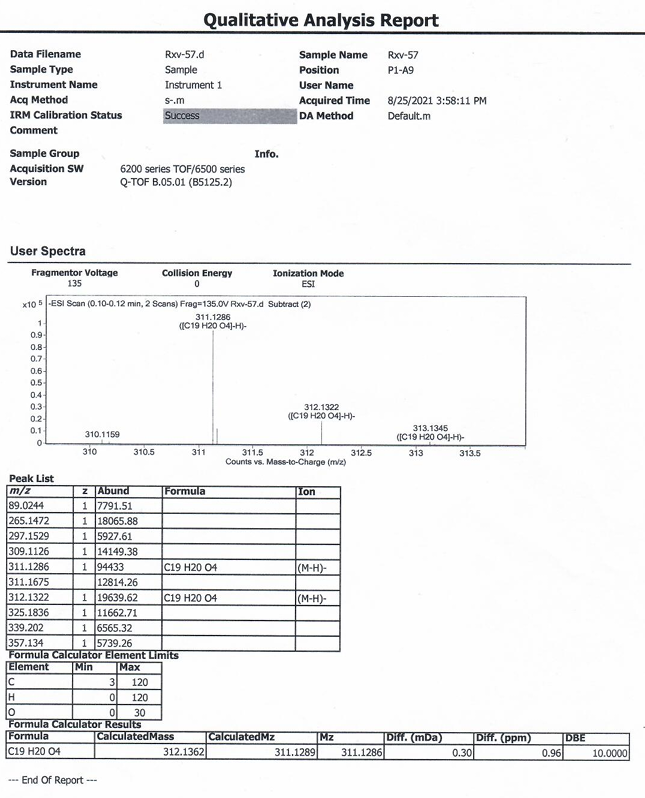


# Figure S35. IR spectrum of 3


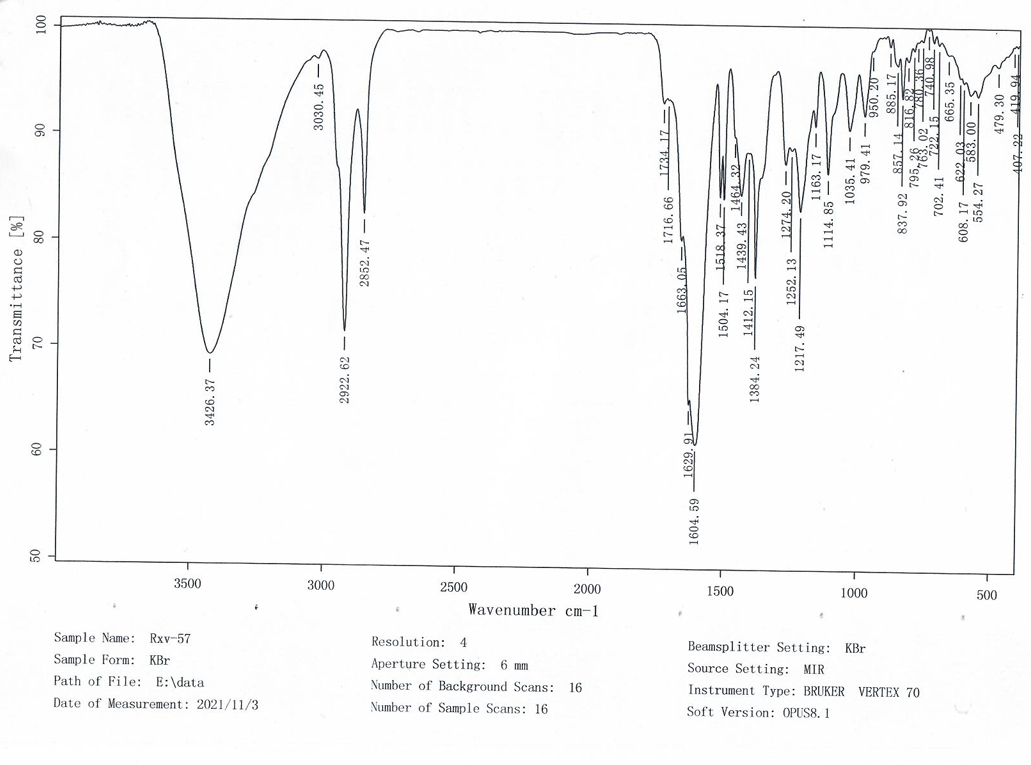


# Figure S36. ECD spectrum of (+)-3


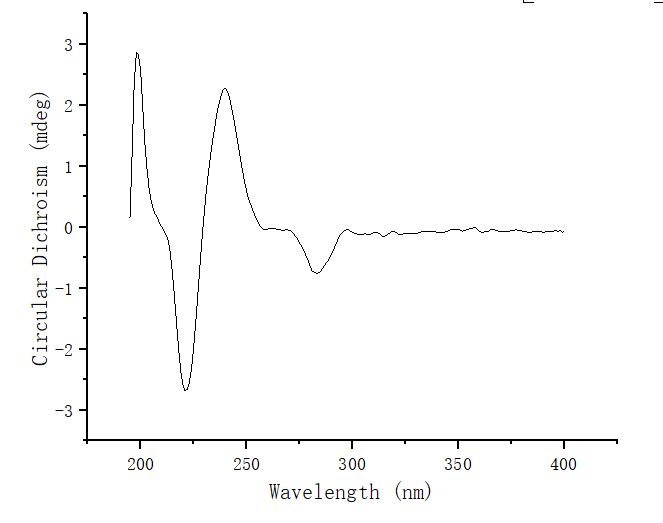


# Figure S37. UV spectrum of (+)-3


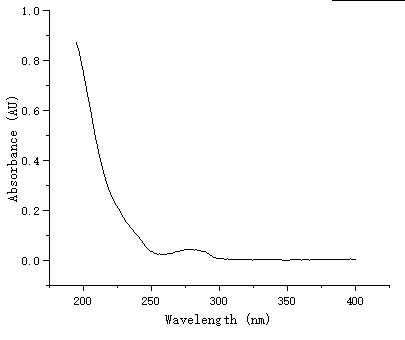


# Figure S38. ECD spectrum of (−)-3


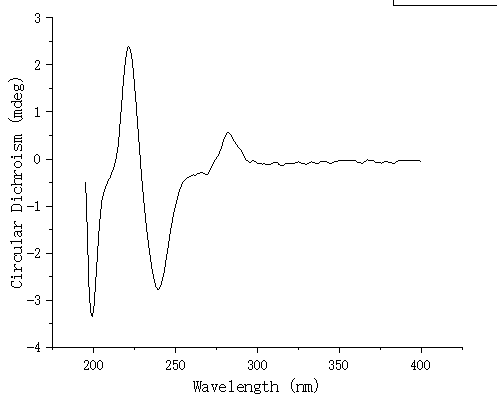


# Figure S39. UV spectrum of (−)-3


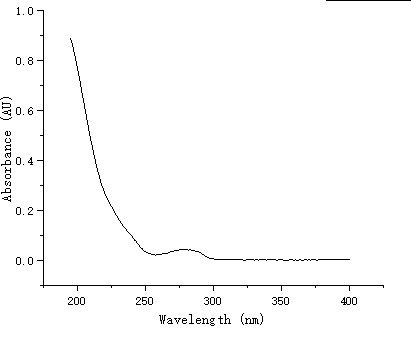


# Figure S40. Individuals of racemic mixtures (±)-4 isolated by chiral resolution


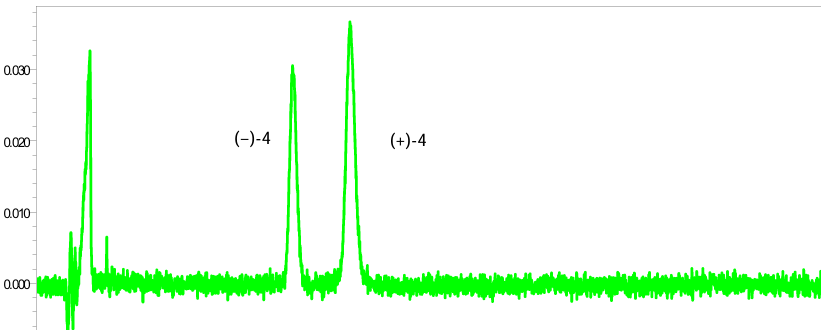


DAICEL CORPORATION semi-preparative column (CNCH_3_/H_2_O, 20:80, *v/v*)

# Figure S41. ^1^H NMR spectrum of 4

# Figure S42. ^13^C NMR spectrum of 4

# Figure S43. HMQC spectrum of 4

# Figure S44. HMBC spectrum of 4

# Figure S45. ^1^H-^1^H COSY spectrum of 4

# **Figure S46.** ROESY spectrum of 4

# Figure S47. HRESIMS spectrum of 4


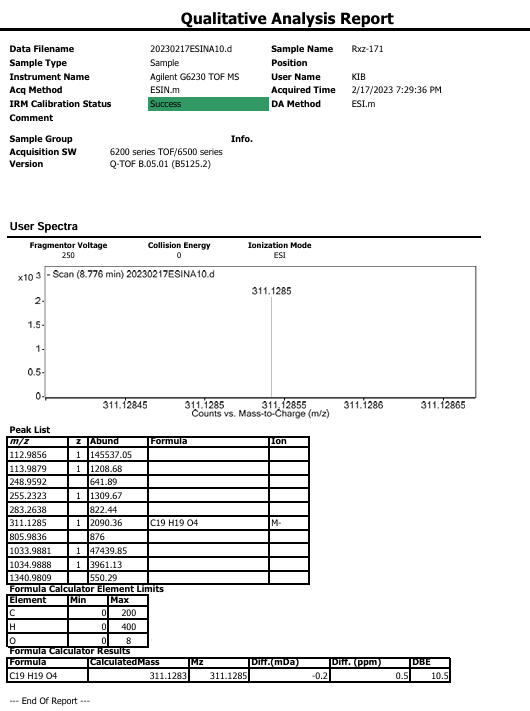


# Figure S48. IR spectrum of 4


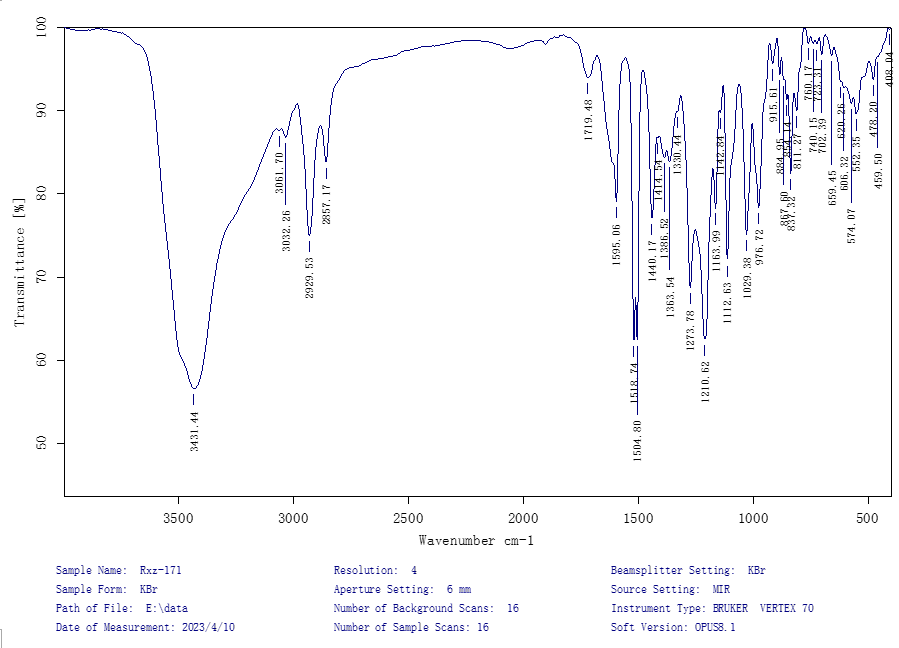


# Figure S49. ECD spectrum of (+)-4


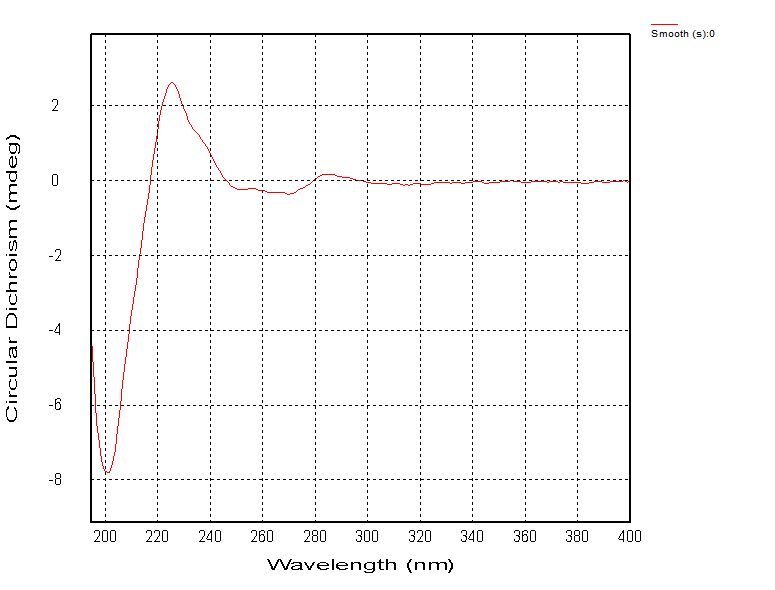


# Figure S50. UV spectrum of (+)-4


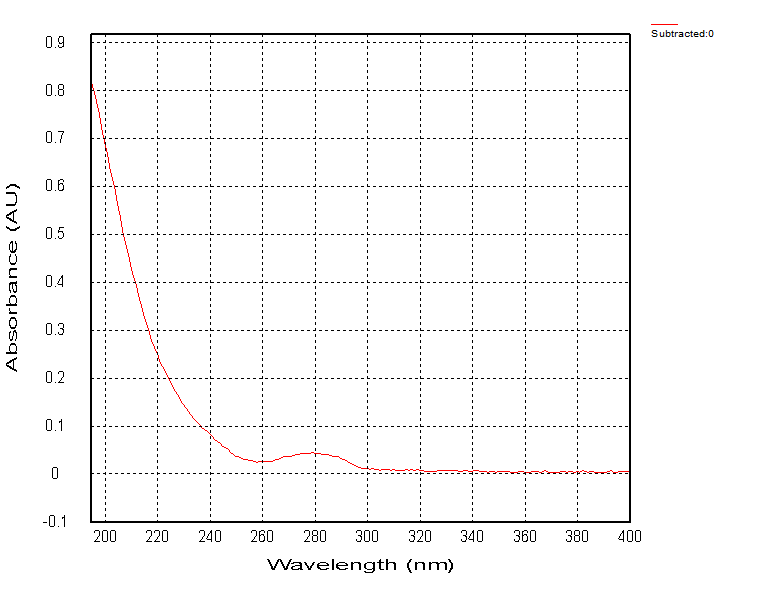


# Figure S51. ECD spectrum of (−)-4


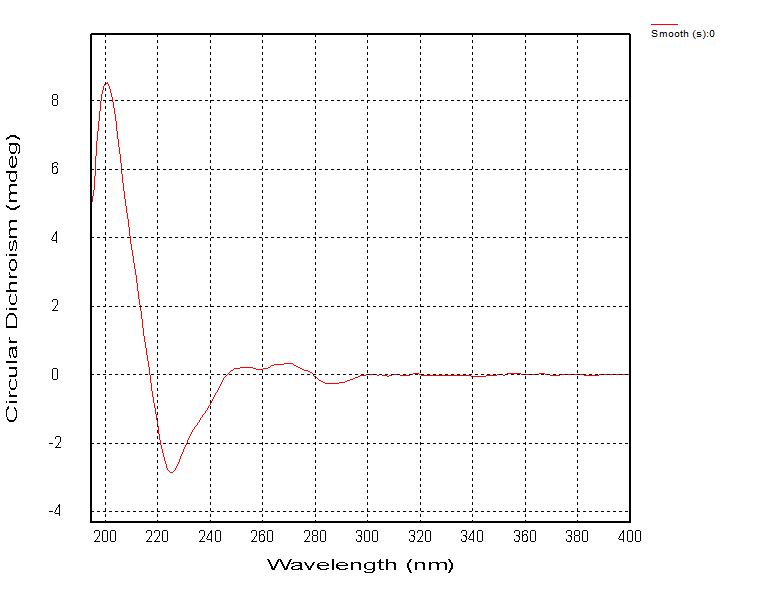


# Figure S52. UV spectrum of (−)-4


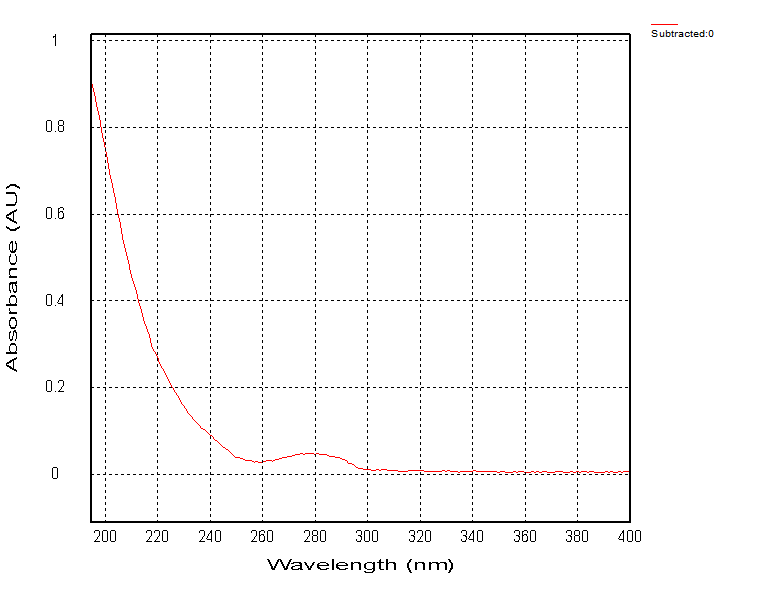


# Figure S53. ^1^H NMR spectrum of 5

# Figure S54. ^13^C NMR spectrum of 5

# Figure S55. HMQC spectrum of 5

# Figure S56. HMBC spectrum of 5

# Figure S57. ^1^H-^1^H COSY spectrum of 5

# Figure 58. ROESY spectrum of 5

# Figure S59. HRESIMS spectrum of 5


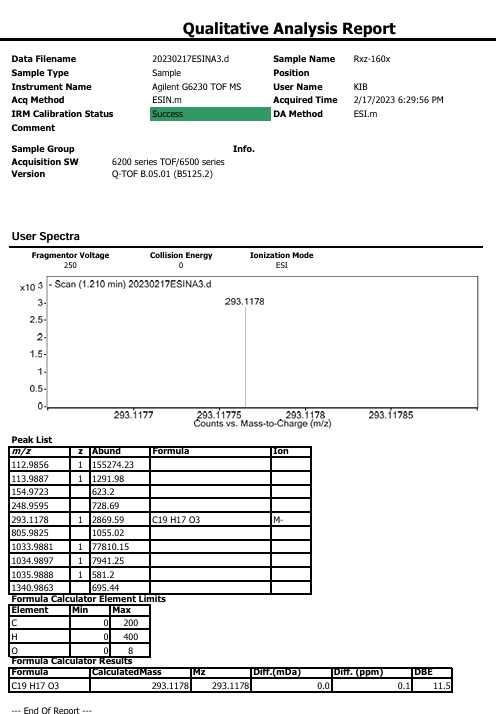


# Figure S60. IR spectrum of 5


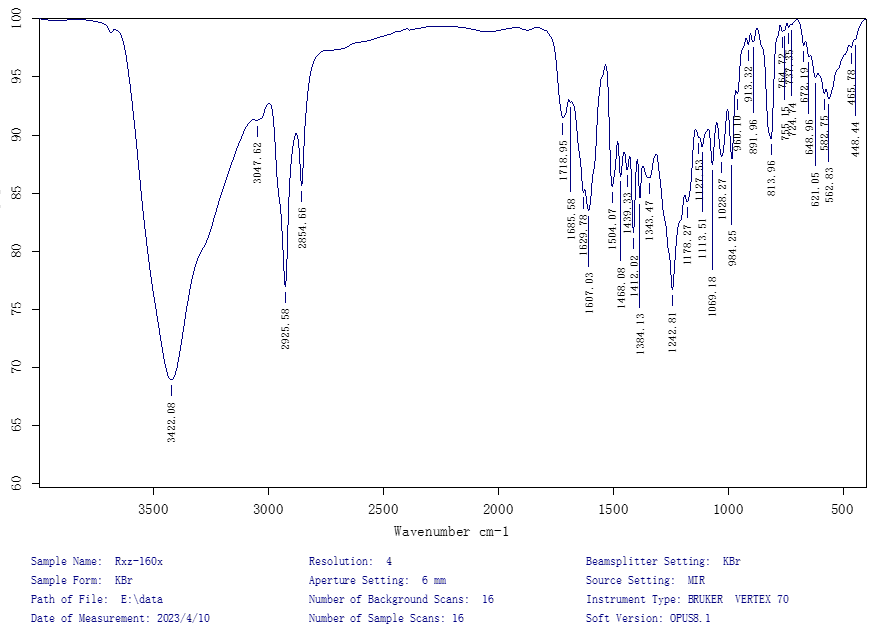


# Figure S61. ECD spectrum of 5


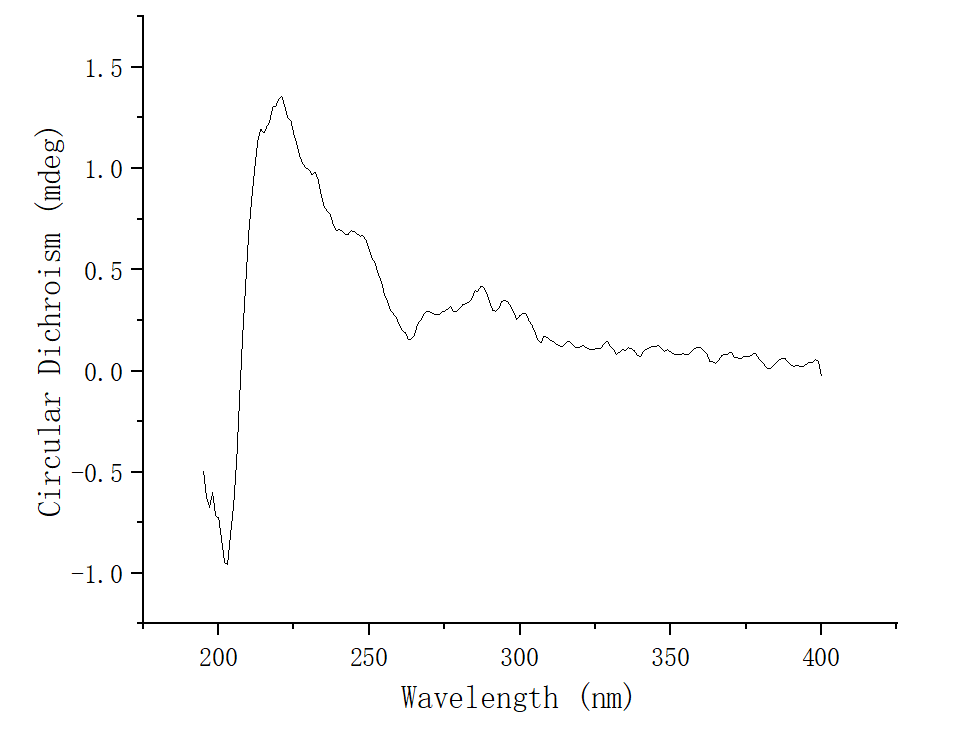


# Figure S62. UV spectrum of 5


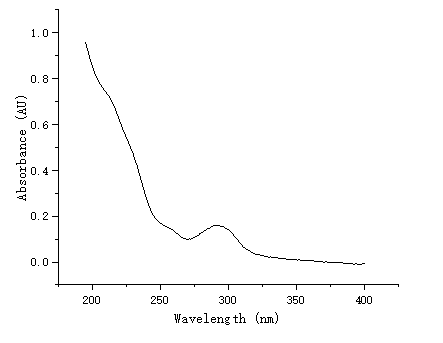


# Figure S63. ^1^H NMR spectrum of 6

# Figure S64. ^13^C NMR spectrum of 6

# Figure S65. HMQC spectrum of 6

# Figure S66. HMBC spectrum of 6

# Figure S67. ^1^H-^1^H COSY spectrum of 6

# Figure S68. ROESY spectrum of 6

# Figure S69. HRESIMS spectrum of 6


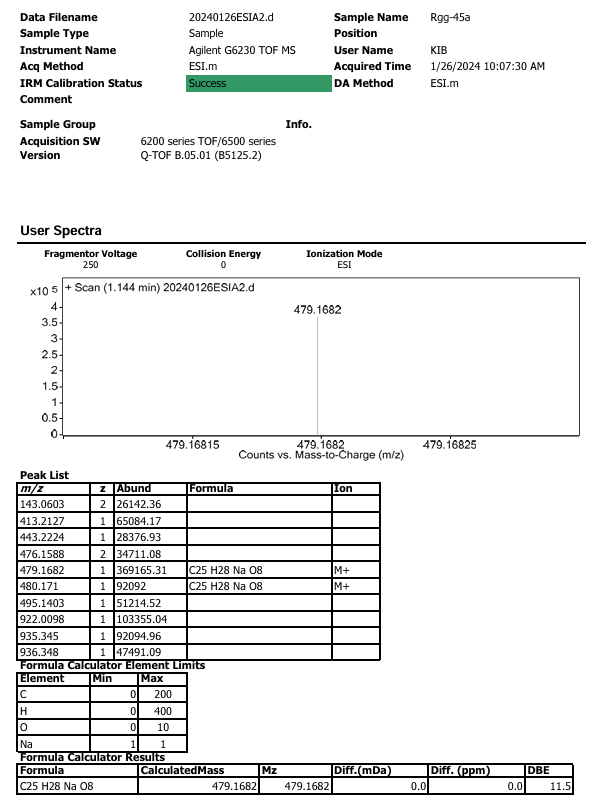


# Figure S70. IR spectrum of 6


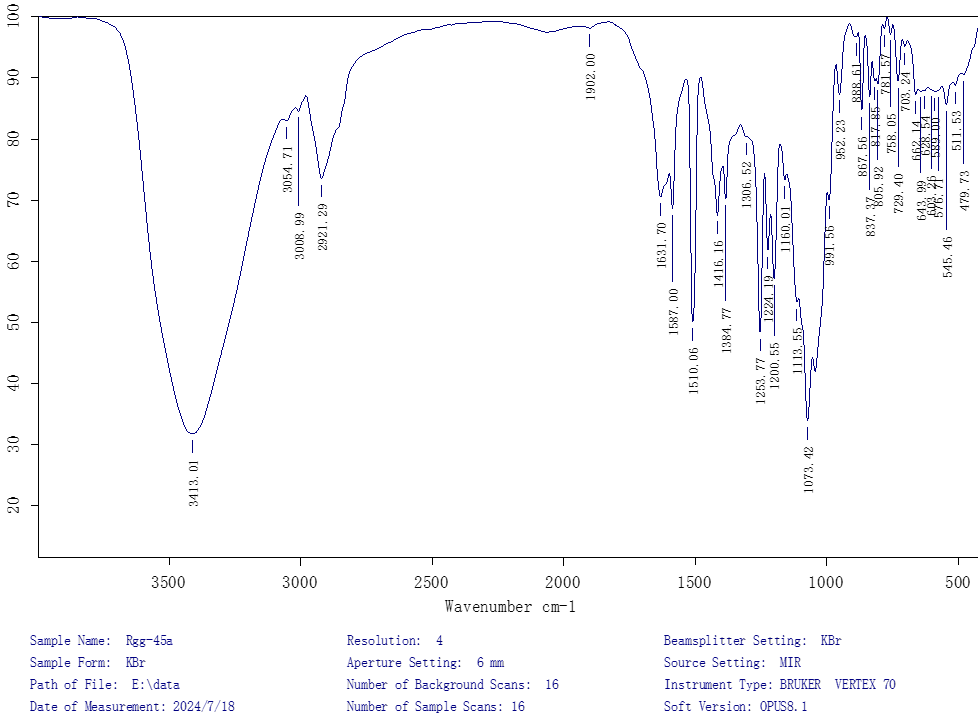


# Figure S71. ECD spectrum of 6


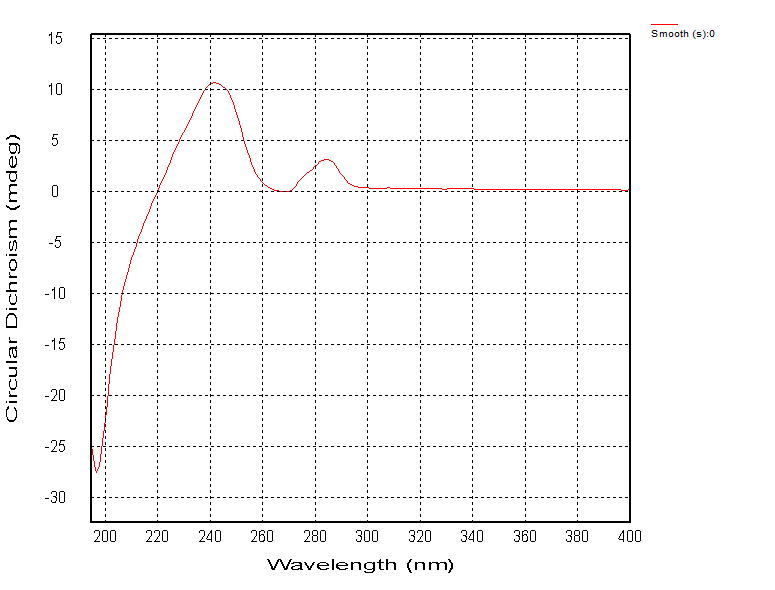


# Figure S72. UV spectrum of 6


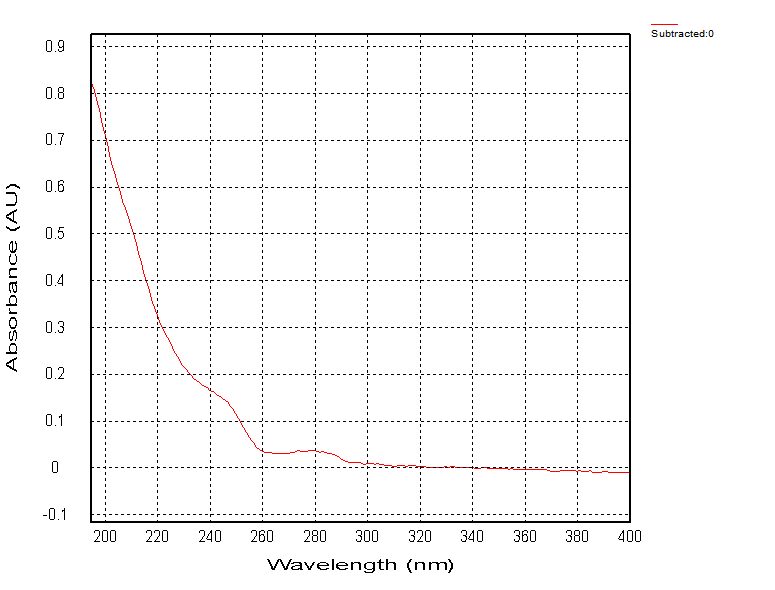


# Figure S73. ^1^H NMR spectrum of synthetic 6

# Figure S74. **^13^C NMR spectrum of** synthetic 6

# Figure S75. HRESIMS spectrum of synthetic 6


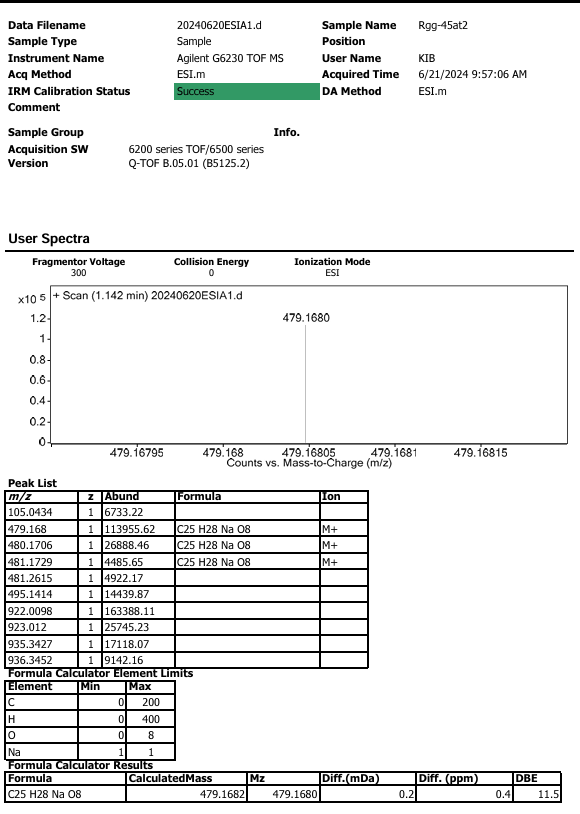


# Figure S76. ECD spectrum of synthetic 6


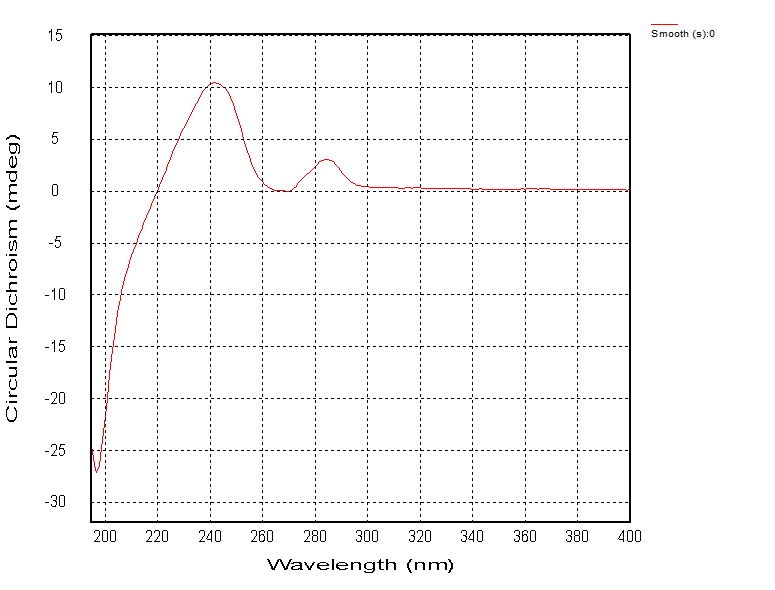


# Figure S77. UV spectrum of synthetic 6


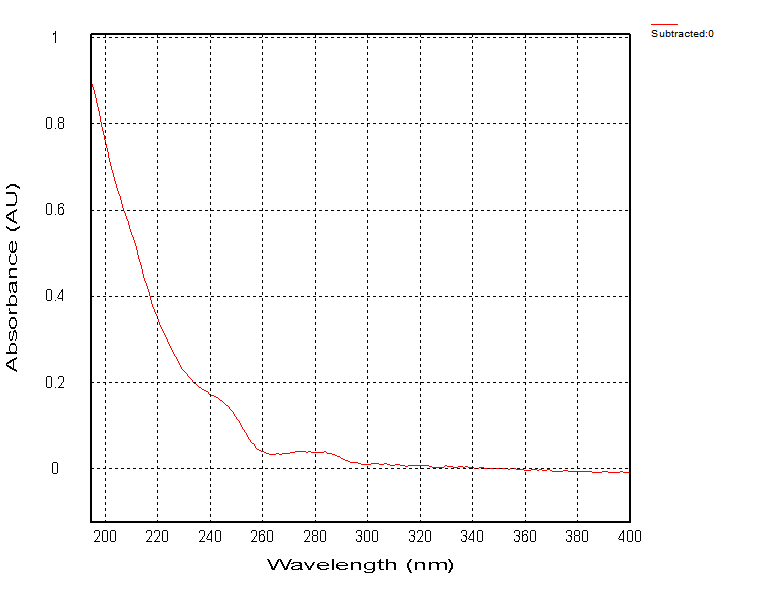


# Figure S78. ^1^H NMR spectrum of 7

# Figure S**79.** ^13^C NMR spectrum of **7**

# Figure S80. HMQC spectrum of 7

# Figure S81. HMBC spectrum of 7

# Figure S82. ^1^H-^1^H COSY spectrum of 7

# Figure S83. ROESY spectrum of 7

# Figure S84. HRESIMS spectrum of 7


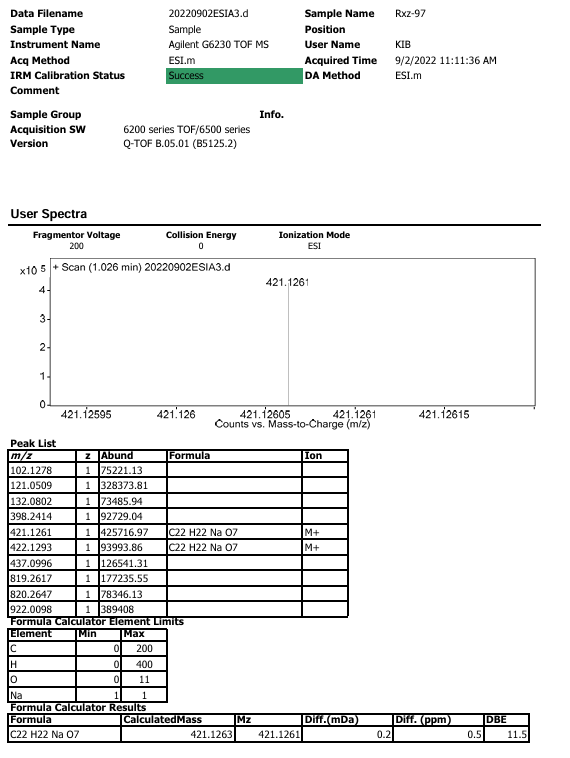


# Figure S85. IR spectrum of 7


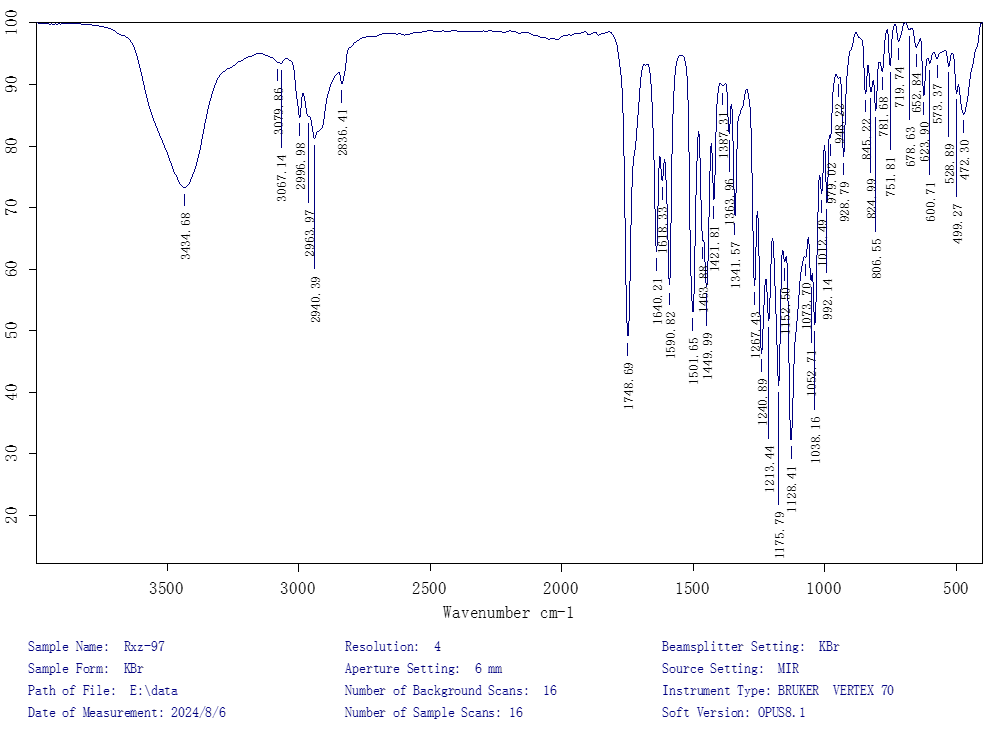


# Figure S86. ECD spectrum of 7


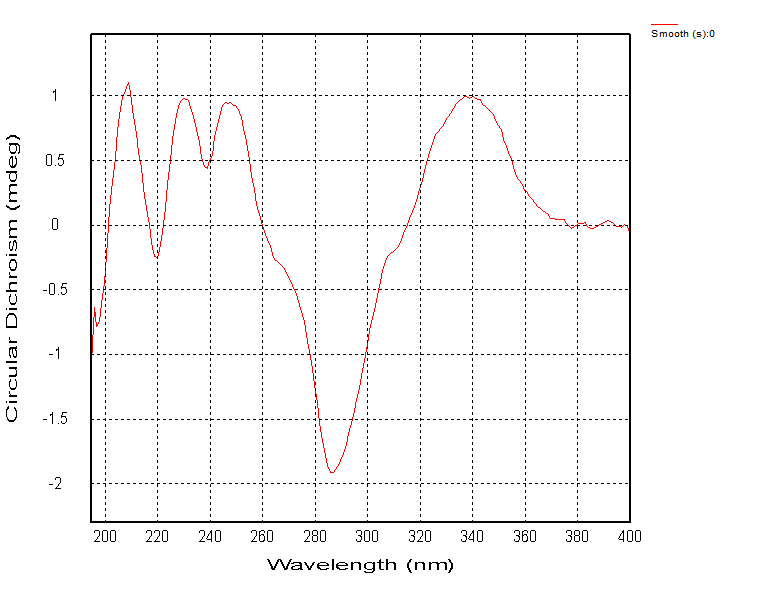


# Figure S87. UV spectrum of 7


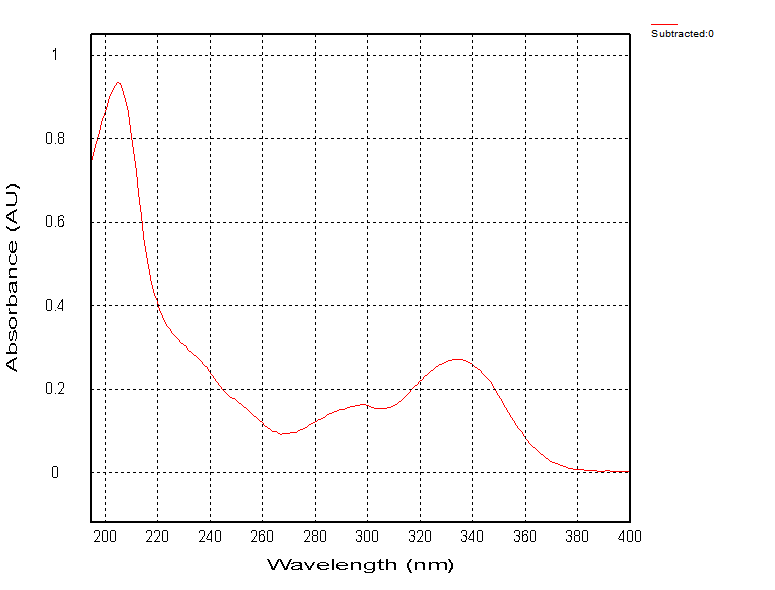


# Figure S88. Inhibitory effects of 1, 2, 4, 6, and 7 against *α*-glucosidase


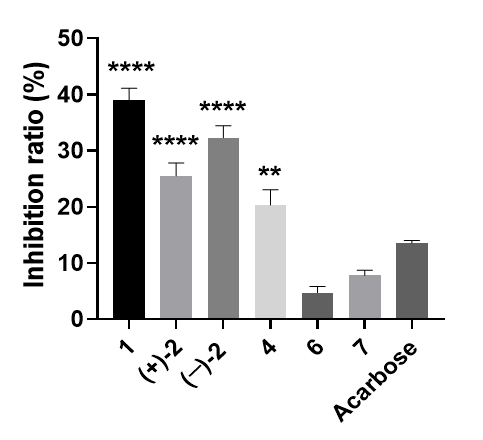


statistical significance was evaluated by the one-way ANOVA test with Tukey multiple comparisons test (MCT). ****P < 0.0001, **P < 0.01 vs. acarbose

# Part 1. X-ray crystal structure of 1

Crystal data for **1**: C_19_H_20_O_4_, *M* = 312.35, *a* = 7.3795(2) Å, *b* = 15.4127(4) Å, *c* = 14.0622(4) Å, *α* = 90°, *β* = 104.6890(10)°, *γ* = 90°, *V* = 1547.13(7) Å^3^, *T* = 150.(2) K, space group *P*121*/c*1, *Z* = 4, *μ*(Cu Kα) = 0.759 mm^-1^, 13446 reflections measured, 2809 independent reflections (*R_int_* = 0.0507). The final *R_1_* values were 0.0424 (*I* > 2*σ*(*I*)). The final *wR*(*F*^2^) values were 0.1080 (*I* > 2*σ*(*I*)). The final *R_1_* values were 0.0462 (all data). The final *wR*(*F*^2^) values were 0.1111 (all data). The goodness of fit on *F*^2^ was 1.040.

**Fig S1-1.** View of a molecule of **1** with the atom-labelling scheme.

Displacement ellipsoids are drawn at the 30% probability level.

**Fig S1-2.** View of the pack drawing of **1**.

Hydrogen-bonds are shown as dashed lines.

**Table S1-1.** Crystal data and structure refinement for **1**_0m.

Identification code global

Empirical formula C19 H20 O4

Formula weight 312.35

Temperature 150(2) K

Wavelength 1.54178 Å

Crystal system Monoclinic

Space group P 1 21/c 1

Unit cell dimensions a = 7.3795(2) Å α= 90°.

b = 15.4127(4) Å β= 104.6890(10)°.

c = 14.0622(4) Å γ = 90°.

Volume 1547.13(7) Å3

Z 4

Density (calculated) 1.341 Mg/m3

Absorption coefficient 0.759 mm-1

F(000) 664

Crystal size 0.570 x 0.440 x 0.360 mm3

Theta range for data collection 4.33 to 68.40°.

Index ranges -8<=h<=7, -18<=k<=18, -16<=l<=16

Reflections collected 13446

Independent reflections 2809 [R(int) = 0.0507]

Completeness to theta = 68.40° 99.0 %

Absorption correction Semi-empirical from equivalents

Max. and min. transmission 0.77 and 0.62

Refinement method Full-matrix least-squares on F2

Data / restraints / parameters 2809 / 0 / 211

Goodness-of-fit on F2 1.040

Final R indices [I>2sigma(I)] R1 = 0.0424, wR2 = 0.1080

R indices (all data) R1 = 0.0462, wR2 = 0.1111

Largest diff. peak and hole 0.270 and -0.257 e.Å-3

# Part 2. X-ray crystal structure of 3

Crystal data for **3**: C_19_H_20_O_4_, *M* = 312.35, *a* = 9.1373(3) Å, *b* = 10.3847(3) Å, *c* = 16.6741(5) Å, *α* = 90°, *β* = 102.2910(10)°, *γ* = 90°, *V* = 1545.91(8) Å^3^, *T* = 150.(2) K, space group *P*121*/c*1, *Z* = 4, *μ*(Cu Kα) = 0.760 mm^-1^, 26920 reflections measured, 3031 independent reflections (*R_int_* = 0.0585). The final *R_1_* values were 0.0376 (*I* > 2*σ*(*I*)). The final *wR*(*F*^2^) values were 0.0913 (*I* > 2*σ*(*I*)). The final *R_1_* values were 0.0404 (all data). The final *wR*(*F*^2^) values were 0.0932 (all data). The goodness of fit on *F*^2^ was 1.024.

**Fig S2-1.** View of a molecule of **3** with the atom-labelling scheme.

Displacement ellipsoids are drawn at the 30% probability level.

**Fig S2-2.** View of the pack drawing of **3**

Hydrogen-bonds are shown as dashed lines.

**Table S2-1.** Crystal data and structure refinement for **3**_0m.

Identification code global

Empirical formula C19 H20 O4

Formula weight 312.35

Temperature 150(2) K

Wavelength 1.54178 Å

Crystal system Monoclinic

Space group P 1 21/c 1

Unit cell dimensions a = 9.1373(3) Å α= 90°.

b = 10.3847(3) Å β= 102.2910(10)°.

c = 16.6741(5) Å γ = 90°.

Volume 1545.91(8) Å3

Z 4

Density (calculated) 1.342 Mg/m3

Absorption coefficient 0.760 mm-1

F(000) 664

Crystal size 0.520 x 0.360 x 0.290 mm3

Theta range for data collection 4.95 to 72.21°.

Index ranges -11<=h<=11, -12<=k<=12, -20<=l<=20

Reflections collected 26920

Independent reflections 3031 [R(int) = 0.0585]

Completeness to theta = 72.21° 99.7 %

Absorption correction Semi-empirical from equivalents

Max. and min. transmission 0.81 and 0.64

Refinement method Full-matrix least-squares on F2

Data / restraints / parameters 3031 / 0 / 211

Goodness-of-fit on F2 1.024

Final R indices [I>2sigma(I)] R1 = 0.0376, wR2 = 0.0913

R indices (all data) R1 = 0.0404, wR2 = 0.0932

Largest diff. peak and hole 0.243 and -0.219 e.Å-3

# Part 3. X-ray crystal structure of 5

Crystal data for **5**: C_19_H_18_O_3_, *M* = 294.33, *a* = 8.9038(4) Å, *b* = 9.4697(4) Å, *c* = 17.1220(7) Å, *α* = 90°, *β* = 90°, *γ* = 90°, *V* = 1443.66(11) Å^3^, *T* = 150.(2) K, space group *Pna*21, *Z* = 4, *μ*(Cu Kα) = 0.729 mm^-1^, 19243 reflections measured, 2586 independent reflections (*R_int_* = 0.1368). The final *R_1_* values were 0.0338 (*I* > 2*σ*(*I*)). The final *wR*(*F*^2^) values were 0.0804 (*I* > 2*σ*(*I*)). The final *R_1_* values were 0.0418 (all data). The final *wR*(*F*^2^) values were 0.0828 (all data). The goodness of fit on *F*^2^ was 1.043. Flack parameter = 0.55(13).

**Fig S3-1.** View of a molecule of **5** with the atom-labelling scheme.

Displacement ellipsoids are drawn at the 30% probability level.

**Fig S3-2.** View of the pack drawing of **5**.

Hydrogen-bonds are shown as dashed lines.

**Table S3-1.** Crystal data and structure refinement for **5**_0m.

Identification code global

Empirical formula C19 H18 O3

Formula weight 294.33

Temperature 150(2) K

Wavelength 1.54178 Å

Crystal system Orthorhombic

Space group Pna2**_1_**

Unit cell dimensions a = 8.9038(4) Å α= 90°.

b = 9.4697(4) Å β= 90°.

c = 17.1220(7) Å γ = 90°.

Volume 1443.66(11) Å3

Z 4

Density (calculated) 1.354 Mg/m3

Absorption coefficient 0.729 mm-1

F(000) 624

Crystal size 0.340 x 0.050 x 0.040 mm3

Theta range for data collection 5.17 to 68.38°.

Index ranges -10<=h<=10, -11<=k<=11, -20<=l<=20

Reflections collected 19243

Independent reflections 2586 [R(int) = 0.1368]

Completeness to theta = 68.38° 99.9 %

Absorption correction Semi-empirical from equivalents

Max. and min. transmission 0.97 and 0.76

Refinement method Full-matrix least-squares on F2

Data / restraints / parameters 2586 / 1 / 202

Goodness-of-fit on F2 1.043

Final R indices [I>2sigma(I)] R1 = 0.0338, wR2 = 0.0804

R indices (all data) R1 = 0.0418, wR2 = 0.0828

Absolute structure parameter 0.55(13)

Largest diff. peak and hole 0.138 and -0.179 e.Å-3

# Part 4. Calculated ECD data of compounds 1

Conformational searching was performed with the Spartan '14 V1.1.4 using Semi-Empirical/PM6. The theoretical calculations were performed using Gaussian 16^1^. Compound **1** were optimized at B3LYP/6-311G (d, p) level. Subsequently, room-temperature (298.15 K) equilibrium populations were calculated according to Boltzmann distribution law:

$$p_{i}=\frac{n_{i}}{\sum_{j} n_{j}}=\frac{e^{-\Delta G_{i}/RT}}{\sum_{j} e^{-\Delta G_{j}/RT}}$$

where $P_{i}$ is the population of the $i^{th}$ conformer; $n_{i}$ the number of molecules in $i^{th}$ conformer; ΔG is the relative Gibbs free energy (kcal/mol); T is room temperature (298.15 K); R is the ideal gas constant (0.0019858995).The theoretical calculation of ECD was performed using time dependent Density Functional Theory (TDDFT) at B3LYP/6-311G (d, p) level in methanol with IEFPCM solvent model. The calculated ECD curve was generated using the Multiwfn software^2^.

Reference:

[1] Frisch MJ, Trucks GW, Schlegel HB, Scuseria GE, Robb MA, Cheeseman JR, *et al*. Gaussian, Inc., Wallingford CT, 2016.

[2] Lu T, Chen FW, Multiwfn: A Multifunctional Wavefunction Analyzer. *J. Comput. Chem* 2012, 33, 580–592.


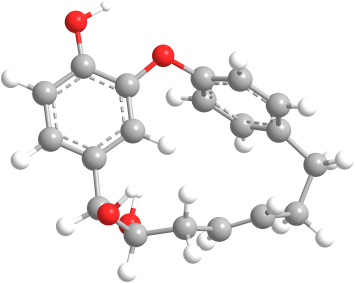

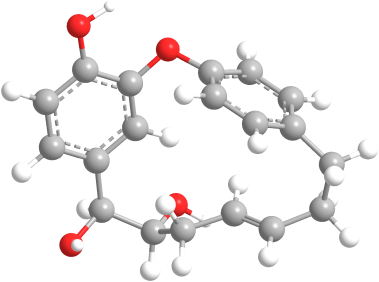

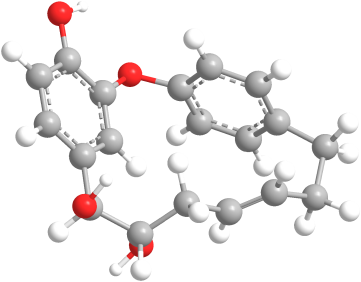


**1-1** **1-2** **1-3**

**Figure S4-1.** Optimized geometries of 3 dominant conformers of **1** at the B3LYP/6-311G(d, p) level of theory in the gas phase.

**Table S4-1.** Conformational analysis of the B3LYP/6-311G(d, p) optimized conformers of **1** in the gas phase (T=298.15 K).

| Conformer | E (Hartree) | G (kcal/mol) | Population |
| --- | --- | --- | --- |
| **1-1** | -1037.1883722 | -650847.430296063 | 19.39% |
| **1-2** | -1037.1866519 | -650847.826443126 | 37.87% |
| **1-3** | -1037.1900932 | -650847.897916515 | 42.73% |

**Table S4-2**. Atomic coordinates (Å) of **1-1** obtained at the B3LYP/6-311G(d, p) level of theory in the gas phase.

| C | 4.059798 | -0.580219 | -0.097768 |  | O | 0.965411 | -2.46967 | 0.039746 |
| --- | --- | --- | --- | --- | --- | --- | --- | --- |
| C | 3.605995 | 0.736546 | -0.062174 |  | H | 5.117346 | -0.80554 | -0.16934 |
| C | 2.242484 | 1.033338 | 0.026932 |  | H | 4.327061 | 1.544954 | -0.10779 |
| C | 1.335388 | -0.036992 | 0.079349 |  | H | 0.272506 | 0.13701 | 0.163071 |
| C | 1.784648 | -1.352304 | 0.022409 |  | H | 2.538115 | 3.053896 | -0.51888 |
| C | 3.15526 | -1.636643 | -0.061011 |  | H | 0.428613 | 3.913643 | -0.68404 |
| C | 1.81706 | 2.497192 | 0.083917 |  | H | -1.00072 | 3.490318 | 0.983373 |
| C | 0.41958 | 2.837721 | -0.500018 |  | H | -0.48113 | 1.856644 | 1.259932 |
| C | -0.775648 | 2.55138 | 0.467455 |  | H | -2.03822 | 2.065566 | -1.29055 |
| C | -2.021633 | 2.016134 | -0.205513 |  | H | -3.03453 | 1.389776 | 1.518617 |
| C | -3.0524 | 1.457077 | 0.43028 |  | H | -4.1086 | 0.903818 | -1.33637 |
| C | -4.230952 | 0.814848 | -0.252704 |  | H | -5.16233 | 1.332605 | 0.006773 |
| O | 1.957867 | 3.051871 | 1.402366 |  | H | 1.534401 | 2.451561 | 2.025973 |
| O | 0.286432 | 2.277117 | -1.808738 |  | H | 0.423741 | 1.324036 | -1.75317 |
| C | -4.403823 | -0.690518 | 0.125142 |  | H | -5.12337 | -1.14178 | -0.56453 |
| C | -3.079877 | -1.417847 | 0.092989 |  | H | -4.83192 | -0.76291 | 1.128734 |
| C | -2.396879 | -1.691735 | 1.282536 |  | H | -2.89414 | -1.53195 | 2.2331 |
| C | -1.063778 | -2.096109 | 1.273711 |  | H | -0.51809 | -2.25176 | 2.196754 |
| C | -0.411425 | -2.230533 | 0.055863 |  | H | -0.57074 | -2.2183 | -2.08345 |
| C | -1.094617 | -2.075344 | -1.14575 |  | H | -2.94793 | -1.50604 | -2.05613 |
| C | -2.428401 | -1.676089 | -1.119024 |  | H | 2.829296 | -3.50637 | -0.08899 |
| O | 3.599783 | -2.921639 | -0.113728 |  |  |  |  |  |

**Table S4-3**. Atomic coordinates (Å) of **1-2** obtained at the B3LYP/6-311G(d, p) level of theory in the gas phase.

| C | -3.889594 | -0.73175 | -0.517225 | O | -0.88038 | -2.43409 | 0.651524 |
| --- | --- | --- | --- | --- | --- | --- | --- |
| C | -3.522601 | 0.605579 | -0.388451 | H | -4.85787 | -1.00791 | -0.91816 |
| C | -2.269737 | 0.970932 | 0.123102 | H | -4.21451 | 1.377333 | -0.70336 |
| C | -1.399201 | -0.050241 | 0.515168 | H | -0.43771 | 0.185035 | 0.944874 |
| C | -1.746435 | -1.384189 | 0.344976 | H | -2.49887 | 2.900931 | 1.030698 |
| C | -2.997489 | -1.743952 | -0.164983 | H | -0.39564 | 3.895148 | 0.551743 |
| C | -1.911656 | 2.454492 | 0.222563 | H | 0.71063 | 3.0791 | -1.29266 |
| C | -0.43743 | 2.801596 | 0.531439 | H | 0.113616 | 1.450111 | -1.08269 |
| C | 0.567468 | 2.291838 | -0.547257 | H | 1.898839 | 1.503612 | 1.0289 |
| C | 1.897779 | 1.818748 | -0.010865 | H | 3.046395 | 1.886034 | -1.77088 |
| C | 3.015026 | 1.623659 | -0.713245 | H | 4.193119 | 0.974046 | 0.94357 |
| C | 4.227111 | 0.926527 | -0.149177 | H | 5.147987 | 1.429377 | -0.46407 |
| O | -2.325968 | 3.161754 | -0.955242 | H | -2.01735 | 2.666663 | -1.72328 |
| O | -0.179218 | 2.307309 | 1.853382 | H | 0.539198 | 2.822801 | 2.232758 |
| C | 4.329741 | -0.574873 | -0.57175 | H | 5.180215 | -1.0191 | -0.04516 |
| C | 3.051012 | -1.322185 | -0.269417 | H | 4.546407 | -0.63646 | -1.64208 |
| C | 2.124223 | -1.57863 | -1.286402 | H | 2.397443 | -1.38683 | -2.31853 |
| C | 0.834146 | -2.009563 | -0.995901 | H | 0.104513 | -2.15252 | -1.78436 |
| C | 0.459417 | -2.183056 | 0.332817 | H | 1.086466 | -2.21957 | 2.381778 |
| C | 1.390036 | -2.049201 | 1.355669 | H | 3.391534 | -1.46825 | 1.852501 |
| C | 2.683019 | -1.625762 | 1.045827 | H | -2.57572 | -3.58775 | -0.07388 |
| O | -3.337898 | -3.051423 | -0.335794 |  |  |  |  |

**Table S4-4**. Atomic coordinates (Å) of **1-3** obtained at the B3LYP/6-311G(d, p) level of theory in the gas phase.

| C | -3.814196 | -0.826691 | -0.61092 | O | -0.82728 | -2.46618 | 0.696736 |
| --- | --- | --- | --- | --- | --- | --- | --- |
| C | -3.485805 | 0.518663 | -0.45979 | H | -4.75932 | -1.12347 | -1.05061 |
| C | -2.26297 | 0.910738 | 0.101752 | H | -4.1874 | 1.275669 | -0.78817 |
| C | -1.377756 | -0.091612 | 0.512151 | H | -0.43667 | 0.169609 | 0.970971 |
| C | -1.69411 | -1.432266 | 0.337257 | H | -2.5538 | 2.754357 | 1.144862 |
| C | -2.916549 | -1.819252 | -0.22035 | H | -0.52408 | 3.901554 | 0.689604 |
| C | -1.970337 | 2.400991 | 0.285675 | H | 0.477804 | 3.339989 | -1.26564 |
| C | -0.501242 | 2.812357 | 0.566354 | H | 0.100938 | 1.63469 | -1.17014 |
| C | 0.478557 | 2.480077 | -0.58722 | H | 2.149599 | 2.449847 | 0.874622 |
| C | 1.889873 | 2.160039 | -0.13896 | H | 2.522012 | 1.182658 | -1.8781 |
| C | 2.78551 | 1.492025 | -0.86656 | H | 4.218203 | 1.294376 | 0.698003 |
| C | 4.126617 | 1.028038 | -0.35933 | H | 4.94456 | 1.534082 | -0.88677 |
| O | -2.476147 | 3.174471 | -0.81115 | H | -2.14803 | 2.78043 | -1.62822 |
| O | -0.032975 | 2.22419 | 1.787226 | H | -0.58901 | 2.545347 | 2.505797 |
| C | 4.340667 | -0.512413 | -0.525 | H | 5.186659 | -0.81444 | 0.099254 |
| C | 3.092792 | -1.291275 | -0.17648 | H | 4.61656 | -0.72402 | -1.56188 |
| C | 2.226638 | -1.709406 | -1.19428 | H | 2.540156 | -1.62697 | -2.22946 |
| C | 0.94295 | -2.160838 | -0.90915 | H | 0.254482 | -2.42498 | -1.70335 |
| C | 0.517804 | -2.200846 | 0.414135 | H | 1.05403 | -1.98396 | 2.478146 |
| C | 1.395848 | -1.913626 | 1.45229 | H | 3.345432 | -1.18119 | 1.959855 |
| C | 2.681671 | -1.463987 | 1.149444 | H | -2.46297 | -3.65314 | -0.09849 |
| O | -3.224215 | -3.13432 | -0.39611 |  |  |  |  |

# Part 5. Calculated ECD data of compounds 2

Conformational searching was performed with the Spartan '14 V1.1.4 using Semi-Empirical/PM6. The theoretical calculations were performed using Gaussian 16^1^. Compound **2** were optimized at B3LYP/6-31G (d, p) level. Subsequently, room-temperature (298.15 K) equilibrium populations were calculated according to Boltzmann distribution law:

$$p_{i}=\frac{n_{i}}{\sum_{j} n_{j}}=\frac{e^{-\Delta G_{i}/RT}}{\sum_{j} e^{-\Delta G_{j}/RT}}$$

where $P_{i}$ is the population of the $i^{th}$ conformer; $n_{i}$ the number of molecules in $i^{th}$ conformer; ΔG is the relative Gibbs free energy (kcal/mol); T is room temperature (298.15 K); R is the ideal gas constant (0.0019858995).The theoretical calculation of ECD was performed using time dependent Density Functional Theory (TDDFT) at B3LYP/6-311G (d, p) level in methanol with IEFPCM solvent model. The calculated ECD curve was generated using the Multiwfn software^2^.


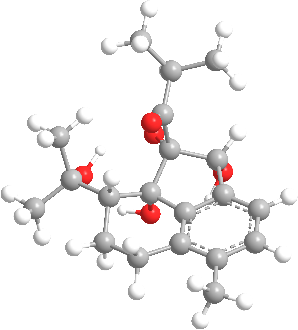

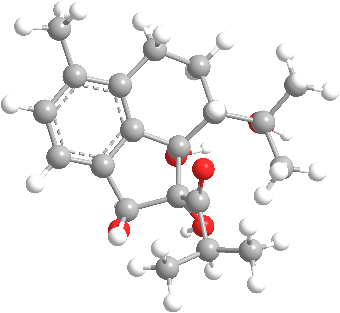


**2-1** **2-2**

**Figure S5-1** Optimized geometries of 2 dominate conformers of **2** at the 6-31G level of theory in the gas phase.

**Table S5-1.** Conformational analysis of the B3LYP/6-31G(d, p) optimized conformers of **2** in the gas phase (T=298.15 K).

| Conformer | E (Hartree) | G (kcal/mol) | Population |
| --- | --- | --- | --- |
| **1-1** | -1037.1972215 | -650851.628463465 | 50.00% |
| **1-2** | -1037.1972215 | -650851.628463465 | 50.00% |

**Table S5-2**. Atomic coordinates (Å) of **2-1** obtained at the B3LYP/6-31G(d, p) level of theory in the gas phase.

| C | 3.810856 | -1.015192 | 0.518396 | O | 0.745828 | -2.606841 | -0.651754 |
| --- | --- | --- | --- | --- | --- | --- | --- |
| C | 3.50226 | 0.3361 | 0.382132 | H | 4.77124 | -1.330887 | 0.908893 |
| C | 2.26017 | 0.748382 | -0.116333 | H | 4.235023 | 1.080875 | 0.667724 |
| C | 1.3333 | -0.236503 | -0.472101 | H | 0.358244 | 0.026999 | -0.855306 |
| C | 1.635046 | -1.584845 | -0.326229 | H | 2.547319 | 2.559601 | -1.211422 |
| C | 2.879516 | -1.991267 | 0.167281 | H | 0.165093 | 2.029121 | -1.455057 |
| C | 1.985291 | 2.231515 | -0.330919 | H | -0.115718 | 1.661959 | 1.252249 |
| C | 0.522789 | 2.625438 | -0.612649 | H | -0.389963 | 3.394449 | 1.167093 |
| C | -0.442776 | 2.466112 | 0.58566 | H | -2.124005 | 2.584282 | -0.849804 |
| C | -1.857962 | 2.189749 | 0.130659 | H | -2.491591 | 1.081015 | 1.781911 |
| C | -2.75741 | 1.466304 | 0.797982 | H | -4.178669 | 1.34519 | -0.785648 |
| C | -4.107966 | 1.058116 | 0.267681 | H | -4.910465 | 1.588531 | 0.794797 |
| O | 2.514682 | 3.047668 | 0.727896 | H | 2.198572 | 2.69202 | 1.567497 |
| O | 0.496648 | 3.972587 | -1.096103 | H | 0.973264 | 4.499273 | -0.440782 |
| C | -4.383844 | -0.474395 | 0.409596 | H | -5.218705 | -0.739127 | -0.245482 |
| C | -3.154905 | -1.296639 | 0.095545 | H | -4.702069 | -0.685177 | 1.434272 |
| C | -2.342191 | -1.758522 | 1.138215 | H | -2.688995 | -1.675479 | 2.162632 |
| C | -1.06643 | -2.25427 | 0.893001 | H | -0.415695 | -2.551498 | 1.706965 |
| C | -0.59872 | -2.297663 | -0.415128 | H | -1.053783 | -2.042939 | -2.494879 |
| C | -1.42804 | -1.968058 | -1.480731 | H | -3.329605 | -1.156574 | -2.046964 |
| C | -2.70504 | -1.471543 | -1.217595 | H | 2.394933 | -3.819416 | 0.060344 |
| O | 3.176829 | -3.310806 | 0.319235 |  |  |  |  |

**Table S5-3**. Atomic coordinates (Å) of **2-2** obtained at the B3LYP/6-31G(d, p) level of theory in the gas phase

| C | -3.81124 | -1.01482 | -0.51788 | O | -0.74606 | -2.60687 | 0.65124 |
| --- | --- | --- | --- | --- | --- | --- | --- |
| C | -3.50244 | 0.336436 | -0.38175 | H | -4.77181 | -1.33042 | -0.90801 |
| C | -2.2601 | 0.74857 | 0.116217 | H | -4.23521 | 1.081302 | -0.66709 |
| C | -1.33322 | -0.23642 | 0.471654 | H | -0.35798 | 0.026968 | 0.854467 |
| C | -1.6352 | -1.58473 | 0.325974 | H | -2.54713 | 2.559887 | 1.211135 |
| C | -2.8799 | -1.991 | -0.16707 | H | -0.16502 | 2.029193 | 1.455076 |
| C | -1.98505 | 2.231703 | 0.330703 | H | 0.116097 | 1.662003 | -1.25223 |
| C | -0.52254 | 2.625526 | 0.612601 | H | 0.390486 | 3.394451 | -1.16702 |
| C | 0.443151 | 2.46611 | -0.58558 | H | 2.124195 | 2.584035 | 0.85013 |
| C | 1.858266 | 2.189602 | -0.1304 | H | 2.492001 | 1.080942 | -1.78167 |
| C | 2.757725 | 1.466129 | -0.79768 | H | 4.178812 | 1.344665 | 0.786111 |
| C | 4.108184 | 1.057745 | -0.26727 | H | 4.910811 | 1.588137 | -0.79421 |
| O | -2.51425 | 3.047812 | -0.72821 | H | -2.19814 | 2.692054 | -1.56776 |
| O | -0.49636 | 3.972688 | 1.095987 | H | -0.97279 | 4.499413 | 0.440564 |
| C | 4.383901 | -0.47478 | -0.40936 | H | 5.218708 | -0.7397 | 0.245712 |
| C | 3.154848 | -1.29692 | -0.09551 | H | 4.702164 | -0.68545 | -1.43405 |
| C | 2.342213 | -1.75866 | -1.1383 | H | 2.68915 | -1.67558 | -2.16267 |
| C | 1.066379 | -2.25431 | -0.89327 | H | 0.415716 | -2.55143 | -1.70733 |
| C | 0.598511 | -2.29776 | 0.414802 | H | 1.053352 | -2.0432 | 2.49462 |
| C | 1.427734 | -1.96828 | 1.48052 | H | 3.329296 | -1.15699 | 2.047031 |
| C | 2.704805 | -1.47185 | 1.217567 | H | -2.39555 | -3.81922 | -0.06029 |
| O | -3.1775 | -3.3105 | -0.31881 |  |  |  |  |

# Part 6. Calculated ECD data of compounds 3

Conformational searching was performed with the Spartan '14 V1.1.4 using Semi-Empirical/PM6. The theoretical calculations were performed using Gaussian 16^1^. Compound **3** were optimized at B3LYP/6-31G (d) level. Subsequently, room-temperature (298.15 K) equilibrium populations were calculated according to Boltzmann distribution law:

$$p_{i}=\frac{n_{i}}{\sum_{j} n_{j}}=\frac{e^{-\Delta G_{i}/RT}}{\sum_{j} e^{-\Delta G_{j}/RT}}$$

where $P_{i}$ is the population of the $i^{th}$ conformer; $n_{i}$ the number of molecules in $i^{th}$ conformer; ΔG is the relative Gibbs free energy (kcal/mol); T is room temperature (298.15 K); R is the ideal gas constant (0.0019858995).The theoretical calculation of ECD was performed using time dependent Density Functional Theory (TDDFT) at B3LYP/6-311G (d, p) level in methanol with IEFPCM solvent model. The calculated ECD curve was generated using the Multiwfn software^2^.


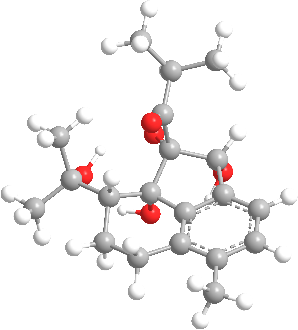

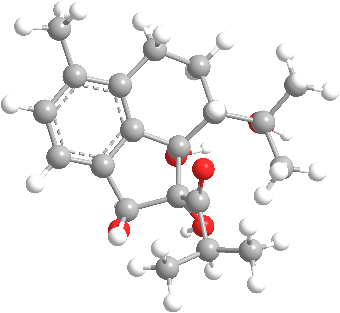

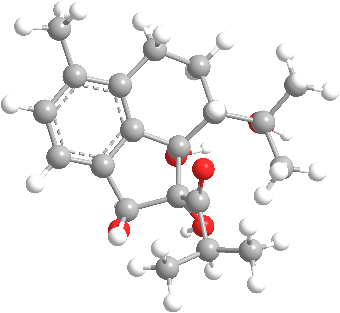


3-1 3-2 3-3

**Figure S6-1** Optimized geometries of 3 dominate conformers of **3** at the 6-31G level of theory in the gas phase.

**Table S6-1.** Conformational analysis of the B3LYP/6-31G(d, p) optimized conformers of **3** in the gas phase (T=298.15 K).

| Conformer | E (Hartree) | G (kcal/mol) | Population |
| --- | --- | --- | --- |
| **1-1** | -1037.1926543 | -650848.762499793 | 34.83% |
| **1-2** | -1037.192575 | -650848.71273825 | 32.02% |
|  | -1037.192608 | -650848.73344608 | 33.16 |

**Table S6-2**. Atomic coordinates (Å) of **3-1** obtained at the B3LYP/6-31G(d, p) level of theory in the gas phase

| C | -4.21329 | 1.037863 | 0.198903 | O | 1.486168 | 3.479037 | -0.65153 |
| --- | --- | --- | --- | --- | --- | --- | --- |
| C | -3.26324 | 2.053629 | 0.056558 | H | -5.25875 | 1.274166 | 0.374648 |
| C | -1.91246 | 1.753457 | -0.16644 | H | -3.58469 | 3.090131 | 0.123037 |
| C | -1.54862 | 0.403793 | -0.24114 | H | -0.51811 | 0.12918 | -0.40156 |
| C | -2.48633 | -0.61273 | -0.08427 | H | -0.69899 | -2.22152 | -2.29067 |
| C | -3.83567 | -0.30485 | 0.1348 | H | 1.786458 | -2.23546 | -2.32696 |
| C | -0.13254 | -2.27481 | -1.36597 | H | 1.844956 | -2.36744 | 1.969295 |
| C | -0.79826 | -2.23635 | -0.14442 | H | -0.6377 | -2.32614 | 1.997088 |
| O | -2.17115 | -1.97032 | -0.11997 | H | 2.548765 | 0.392236 | -0.88952 |
| C | 1.2636 | -2.27167 | -1.37451 | H | 4.962068 | -0.43546 | 0.077163 |
| C | 2.000734 | -2.24009 | -0.18203 | H | 3.989203 | -0.74279 | 1.505342 |
| C | 1.299 | -2.34306 | 1.029493 | H | 4.037986 | -2.79463 | 0.290265 |
| C | -0.09583 | -2.33145 | 1.056191 | H | 3.821434 | -2.00887 | -1.2656 |
| C | 3.079492 | 0.588984 | 0.054239 | H | -1.04121 | 3.392862 | -1.27402 |
| C | 3.940003 | -0.64053 | 0.414382 | H | -1.00288 | 3.599332 | 0.468588 |
| C | 3.49522 | -1.98796 | -0.21937 | H | 0.747209 | 1.691076 | -1.15802 |
| C | -0.87229 | 2.8559 | -0.3315 | H | 0.405553 | 1.990316 | 1.853392 |
| C | 0.592525 | 2.384709 | -0.32568 | H | 2.318161 | 0.605128 | 2.143708 |
| C | 1.007137 | 1.739555 | 0.979707 | H | -4.30236 | -2.14545 | 0.24622 |
| C | 2.079104 | 0.952293 | 1.139014 | H | 3.39049 | 2.467428 | -0.33515 |
| O | -4.76845 | -1.29184 | 0.293148 | H | 1.455261 | 4.108016 | 0.090173 |
| O | 3.971854 | 1.705342 | -0.14231 |  |  |  |  |

**Table S6-3**. Atomic coordinates (Å) of **3-2** obtained at the B3LYP/6-31G(d, p) level of theory in the gas phase.

| C | -4.17361 | 1.039756 | 0.314208 | O | 1.500188 | 3.472585 | -0.68132 |
| --- | --- | --- | --- | --- | --- | --- | --- |
| C | -3.23048 | 2.054726 | 0.126158 | H | -5.19847 | 1.275684 | 0.585696 |
| C | -1.90513 | 1.755932 | -0.21756 | H | -3.53663 | 3.089953 | 0.255186 |
| C | -1.55904 | 0.407576 | -0.36691 | H | -0.54829 | 0.132341 | -0.62588 |
| C | -2.48501 | -0.60882 | -0.15288 | H | -0.62974 | -2.26725 | -2.35162 |
| C | -3.80995 | -0.30213 | 0.185513 | H | 1.855759 | -2.28359 | -2.30116 |
| C | -0.09578 | -2.30131 | -1.40686 | H | 1.76514 | -2.32894 | 1.996255 |
| C | -0.80309 | -2.23534 | -0.21041 | H | -0.71619 | -2.28334 | 1.937088 |
| O | -2.17583 | -1.96632 | -0.23944 | H | 2.562567 | 0.380448 | -0.85954 |
| C | 1.300399 | -2.29997 | -1.36673 | H | 4.94784 | -0.43699 | 0.183312 |
| C | 1.995808 | -2.24414 | -0.15061 | H | 3.934394 | -0.73096 | 1.585783 |
| C | 1.252141 | -2.32181 | 1.037793 | H | 4.015508 | -2.7948 | 0.395957 |
| C | -0.14226 | -2.30757 | 1.015648 | H | 3.852066 | -2.02643 | -1.17504 |
| C | 3.066727 | 0.586629 | 0.096862 | H | -1.02155 | 3.34849 | -1.3899 |
| C | 3.91642 | -0.63933 | 0.492914 | H | -1.01332 | 3.631036 | 0.343198 |
| C | 3.490904 | -1.99343 | -0.14062 | H | 0.775509 | 1.681705 | -1.1898 |
| C | -0.86837 | 2.854214 | -0.42136 | H | 0.344586 | 2.006678 | 1.806944 |
| C | 0.596282 | 2.382944 | -0.36897 | H | 2.246063 | 0.621651 | 2.163451 |
| C | 0.970707 | 1.7482 | 0.953068 | H | -4.26778 | -2.14277 | 0.314385 |
| C | 2.036193 | 0.960595 | 1.149431 | H | 3.389712 | 2.46119 | -0.30167 |
| O | -4.72977 | -1.28973 | 0.401507 | H | 1.450804 | 4.109352 | 0.052695 |
| O | 3.964752 | 1.700494 | -0.08544 |  |  |  |  |

**Table S6-4**. Atomic coordinates (Å) of **3-3** obtained at the B3LYP/6-31G(d, p) level of theory in the gas phase.

| C | -4.24736 | 1.02097 | -0.10209 | O | 1.368692 | 3.57067 | -0.48181 |
| --- | --- | --- | --- | --- | --- | --- | --- |
| C | -3.29492 | 2.044 | -0.11806 | H | -5.30711 | 1.245944 | -0.18042 |
| C | -1.92498 | 1.759524 | -0.02393 | H | -3.62933 | 3.074344 | -0.21199 |
| C | -1.54779 | 0.41666 | 0.089493 | H | -0.50469 | 0.155348 | 0.178845 |
| C | -2.48947 | -0.60819 | 0.089278 | H | -0.86755 | -2.06028 | -2.12034 |
| C | -3.85604 | -0.31622 | -0.00434 | H | 1.60388 | -2.0898 | -2.36402 |
| C | -0.22816 | -2.17765 | -1.25078 | H | 2.015481 | -2.50923 | 1.894561 |
| C | -0.79227 | -2.22183 | 0.021898 | H | -0.4575 | -2.45975 | 2.130976 |
| O | -2.15667 | -1.95962 | 0.178401 | H | 2.45069 | 0.390088 | -0.96034 |
| C | 1.16084 | -2.18341 | -1.37564 | H | 4.95479 | -0.4225 | -0.24016 |
| C | 1.994479 | -2.23927 | -0.24862 | H | 4.128273 | -0.74694 | 1.277088 |
| C | 1.394163 | -2.41932 | 1.006876 | H | 4.060696 | -2.79585 | 0.074786 |
| C | 0.005051 | -2.40323 | 1.150336 | H | 3.730996 | -2.01523 | -1.46469 |
| C | 3.058127 | 0.580574 | -0.07045 | H | -1.092 | 3.534948 | -0.9082 |
| C | 3.973338 | -0.63856 | 0.196452 | H | -0.96543 | 3.497321 | 0.839847 |
| C | 3.482451 | -1.99192 | -0.39758 | H | 0.641961 | 1.709926 | -1.04085 |
| C | -0.88309 | 2.87075 | -0.06028 | H | 0.593753 | 1.983834 | 1.992934 |
| C | 0.574651 | 2.407698 | -0.19183 | H | 2.490896 | 0.548282 | 2.080055 |
| C | 1.102334 | 1.735032 | 1.061359 | H | -4.3197 | -2.1607 | 0.033032 |
| C | 2.170733 | 0.928296 | 1.110501 | H | 4.335055 | 2.034773 | 0.328824 |
| O | -4.79415 | -1.31141 | -0.00697 | H | 2.28754 | 3.239477 | -0.53984 |
| O | 3.824201 | 1.758174 | -0.45159 |  |  |  |  |

# Part 7. Calculated ECD data of compounds 4

Conformational searching was performed with the Spartan '14 V1.1.4 using Semi-Empirical/PM6. The theoretical calculations were performed using Gaussian 16^1^. Compound **4** were optimized at B3LYP/6-31G (d, p) level. Subsequently, room-temperature (298.15 K) equilibrium populations were calculated according to Boltzmann distribution law:

$$p_{i}=\frac{n_{i}}{\sum_{j} n_{j}}=\frac{e^{-\Delta G_{i}/RT}}{\sum_{j} e^{-\Delta G_{j}/RT}}$$

where $P_{i}$ is the population of the $i^{th}$ conformer; $n_{i}$ the number of molecules in $i^{th}$ conformer; ΔG is the relative Gibbs free energy (kcal/mol); T is room temperature (298.15 K); R is the ideal gas constant (0.0019858995).The theoretical calculation of ECD was performed using time dependent Density Functional Theory (TDDFT) at B3LYP/6-311G (d, p) level in methanol with IEFPCM solvent model. The calculated ECD curve was generated using the Multiwfn software^2^.


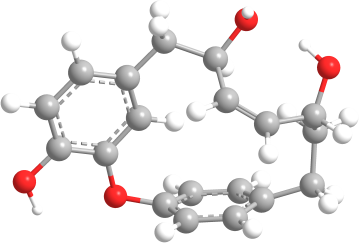

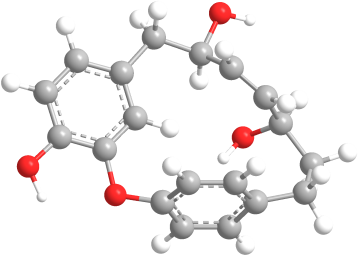

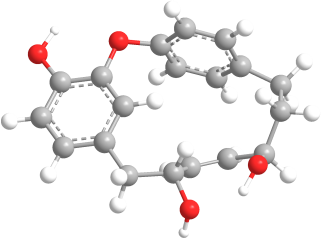


**4-1** **4-2** **4-3**

**Figure S7-1.** Optimized geometries of 3 dominant conformers of **4** at the B3LYP/6-31G(d, p) level of theory in the gas phase.

**Table S7-1.** Conformational analysis of the B3LYP/6-31G(d, p) optimized conformers of **4** in the gas phase (T=298.15 K).

| Conformer | E (Hartree) | G (kcal/mol) | Population |
| --- | --- | --- | --- |
| **4-1** | -1037.1883722 | -650846.075439222 | 13.59% |
| **4-2** | -1037.1866519 | -650844.995933769 | 2.19% |
| **4-3** | -1037.1900932 | -650847.155383932 | 84.22% |

**Table S7-2**. Atomic coordinates (Å) of **4-1** obtained at the B3LYP/6-311G(d, p) level of theory in the gas phase.

| C | -4.05701 | 1.44604 | 0.166463 |  | O | 1.884626 | 3.212341 | -0.6046 |
| --- | --- | --- | --- | --- | --- | --- | --- | --- |
| C | -2.97924 | 2.326745 | 0.040762 |  | H | -5.06116 | 1.817459 | 0.349154 |
| C | -1.68025 | 1.851684 | -0.189 |  | H | -3.15793 | 3.395569 | 0.128669 |
| C | -1.50022 | 0.468258 | -0.29142 |  | H | -0.5139 | 0.064796 | -0.4626 |
| C | -2.56568 | -0.41693 | -0.13939 |  | H | -0.90905 | -2.39562 | -2.24723 |
| C | -3.86136 | 0.06562 | 0.085964 |  | H | 1.569662 | -2.55959 | -2.16668 |
| C | -0.38513 | -2.40968 | -1.29631 |  | H | 1.443288 | -2.33624 | 2.122954 |
| C | -1.09591 | -2.22369 | -0.11253 |  | H | -1.03464 | -2.16214 | 2.034302 |
| O | -2.43005 | -1.80508 | -0.17779 |  | H | 4.349927 | 0.171969 | 1.234012 |
| C | 1.005775 | -2.4885 | -1.23951 |  | H | 2.882908 | -0.43831 | -1.36449 |
| C | 1.691178 | -2.37675 | -0.01872 |  | H | 4.566018 | -0.74939 | -0.96875 |
| C | 0.937055 | -2.36336 | 1.161478 |  | H | 3.601876 | -2.28235 | 0.98324 |
| C | -0.45618 | -2.27947 | 1.122899 |  | H | 3.675723 | -2.86348 | -0.67366 |
| C | 3.597485 | 0.450655 | 0.482868 |  | H | -0.59689 | 3.39518 | -1.2431 |
| C | 3.556084 | -0.7132 | -0.54315 |  | H | -0.54886 | 3.541713 | 0.504467 |
| C | 3.185581 | -2.13094 | -0.02062 |  | H | 0.994792 | 1.516335 | -1.1865 |
| C | -0.50464 | 2.812434 | -0.3176 |  | H | 0.447759 | 1.480686 | 1.720888 |
| C | 0.902528 | 2.183429 | -0.32234 |  | H | 2.305232 | 0.238216 | 2.242785 |
| C | 1.222899 | 1.430957 | 0.957628 |  | H | -4.56582 | -1.69743 | 0.194681 |
| C | 2.313569 | 0.707771 | 1.25982 |  | H | 3.380525 | 2.195608 | -0.43036 |
| O | -4.91632 | -0.79001 | 0.239553 |  | H | 1.929955 | 3.792561 | 0.175689 |
| O | 4.121912 | 1.611241 | -0.16607 |  |  |  |  |  |

**Table S7-3**. Atomic coordinates (Å) of **4-2** obtained at the B3LYP/6-311G(d, p) level of theory in the gas phase.

| C | -4.00794 | 0.623774 | -0.62097 |  | O | 0.746252 | 4.201319 | 0.123178 |
| --- | --- | --- | --- | --- | --- | --- | --- | --- |
| C | -3.2057 | 1.733709 | -0.33946 |  | H | -4.98239 | 0.742258 | -1.08561 |
| C | -1.94356 | 1.590804 | 0.252379 |  | H | -3.57402 | 2.725927 | -0.58726 |
| C | -1.50551 | 0.2941 | 0.55448 |  | H | -0.53609 | 0.151726 | 1.015425 |
| C | -2.29009 | -0.81579 | 0.253994 |  | H | -0.52221 | -2.5281 | -1.72923 |
| C | -3.55426 | -0.66345 | -0.3338 |  | H | 1.943867 | -2.3061 | -1.9287 |
| C | 0.095851 | -2.41125 | -0.84411 |  | H | 2.237531 | -1.89026 | 2.339635 |
| C | -0.5061 | -2.30188 | 0.409744 |  | H | -0.22474 | -2.1682 | 2.534644 |
| O | -1.89115 | -2.13589 | 0.491993 |  | H | 3.401076 | 1.766929 | -1.21179 |
| C | 1.480601 | -2.27588 | -0.94602 |  | H | 3.96059 | -0.55687 | -1.87457 |
| C | 2.262574 | -1.99605 | 0.188176 |  | H | 4.888173 | 0.149704 | -0.5673 |
| C | 1.643999 | -2.05308 | 1.443778 |  | H | 4.074748 | -1.32038 | 1.074908 |
| C | 0.262377 | -2.21507 | 1.56557 |  | H | 4.336481 | -2.31428 | -0.34278 |
| C | 2.872164 | 0.904733 | -0.78331 |  | H | -1.56662 | 3.690594 | 0.149648 |
| C | 3.90224 | -0.2606 | -0.8212 |  | H | -1.0778 | 2.970086 | 1.673096 |
| C | 3.69593 | -1.5216 | 0.066734 |  | H | 0.408725 | 2.438102 | -0.94857 |
| C | -1.09447 | 2.803495 | 0.585939 |  | H | 1.204455 | 2.238612 | 2.02453 |
| C | 0.366035 | 2.804628 | 0.081397 |  | H | 3.03993 | 0.946316 | 1.417823 |
| C | 1.340315 | 2.044487 | 0.958815 |  | H | -3.79823 | -2.54304 | -0.41571 |
| C | 2.400129 | 1.295939 | 0.607801 |  | H | 1.212051 | 0.01704 | -1.31956 |
| O | -4.32348 | -1.75146 | -0.63292 |  | H | 1.68761 | 4.242995 | -0.1119 |
| O | 1.793302 | 0.69362 | -1.70555 |  |  |  |  |  |

**Table S7-4**. Atomic coordinates (Å) of **4-3** obtained at the B3LYP/6-311G(d, p) level of theory in the gas phase.

| C | -4.16164 | 1.201899 | -0.00532 | O | 1.625211 | 3.540535 | -0.24397 |
| --- | --- | --- | --- | --- | --- | --- | --- |
| C | -3.14863 | 2.164134 | -0.00373 | H | -5.20668 | 1.492662 | 0.05016 |
| C | -1.79738 | 1.794861 | -0.07094 | H | -3.4198 | 3.215296 | 0.057626 |
| C | -1.49733 | 0.43018 | -0.15313 | H | -0.46855 | 0.107241 | -0.20825 |
| C | -2.50224 | -0.53538 | -0.13049 | H | -0.66191 | -2.38702 | -2.1937 |
| C | -3.84949 | -0.15774 | -0.06043 | H | 1.813787 | -2.47459 | -2.05107 |
| C | -0.16292 | -2.39167 | -1.22942 | H | 1.571397 | -2.30343 | 2.23667 |
| C | -0.91157 | -2.24989 | -0.06303 | H | -0.90717 | -2.22054 | 2.085667 |
| O | -2.26408 | -1.9093 | -0.16002 | H | 4.381657 | 0.774566 | 0.85781 |
| C | 1.227145 | -2.42856 | -1.13687 | H | 3.142152 | -0.62413 | -1.4942 |
| C | 1.877979 | -2.30979 | 0.102251 | H | 4.779201 | -0.72322 | -0.87229 |
| C | 1.092399 | -2.33635 | 1.261322 | H | 3.726899 | -2.02127 | 1.180485 |
| C | -0.30203 | -2.30096 | 1.187738 | H | 3.914498 | -2.82631 | -0.36836 |
| C | 3.517861 | 0.652174 | 0.177403 | H | -0.87239 | 3.594093 | -0.81796 |
| C | 3.725604 | -0.6949 | -0.57031 | H | -0.79702 | 3.414313 | 0.924003 |
| C | 3.368063 | -2.0242 | 0.144228 | H | 0.895272 | 1.881909 | -1.10986 |
| C | -0.70531 | 2.858146 | -0.02176 | H | 0.494016 | 1.414993 | 1.825483 |
| C | 0.753731 | 2.381701 | -0.14767 | H | 2.429081 | 0.157526 | 1.994253 |
| C | 1.18794 | 1.472118 | 0.987582 | H | -4.42138 | -1.97169 | -0.05869 |
| C | 2.309432 | 0.741269 | 1.082104 | H | 3.144062 | 2.465784 | -0.50779 |
| O | -4.84487 | -1.09513 | -0.03819 | H | 1.648996 | 3.957337 | 0.634869 |
| O | 3.569246 | 1.64817 | -0.84059 |  |  |  |  |

# Part 8. General Procedure of (8*S*)-otteacumiene A-*O*-*β*-D-glucopyranoside

A 5 mL round bottom flask containing substrate (0.0068 mmol, 1.0 equiv.), *α*-D-fluoroglucose (3.72 mg, 0.0204 mmol, 3.0 equiv.), Ca(OH)_2_ (1.51 mg, 0.0204 mmol, 3.0 equiv.), and a magnetic stir bar was charged with H_2_O (50 *µ*L). The flask was sealed with a septum, and the mixture was stirred vigorously at rt for 1 h. Then the mixture was filtered through diatomaceous earth and washed with methanol. Subsequently, it was purified using a semi-preparative HPLC column. Fractions containing the product were concentrated in vacuo to dryness to yield **synthetic 6**, a white powder (0.6 mg, 19 %).

**Synthetic 6:**^1^H NMR (600 MHz, Methanol-*d*_4_): *δ*_H_ 7.35 (1H, dd, *J* = 8.2, 1.8 Hz), 7.30 (1H, dd, *J* = 8.2, 1.8 Hz), 7.25(1H, dd, *J* = 8.2, 1.8 Hz), 7.07 (1H, d, *J* = 2.6 Hz), 7.06 (1H, d, *J* = 8.3 Hz), 6.68 (1H, dd, *J* = 8.3, 1.9 Hz), 6.07 (1H, dt, *J* = 15.3, 4.2 Hz), 5.90 (1H, t, *J* = 11.2 Hz), 5.46 (1H, d, *J* = 1.9 Hz), 5.40 (1H, d, *J* = 11.2 Hz), 5.35 (1H, m), 5.00 (1H, d, *J* = 7.7 Hz), 4.23 (1H, t, *J* = 9.3 Hz), 3.90 (1H, d, *J* =11.9 Hz), 3.73 (1H, dd, *J* = 11.7, 4.3 Hz), 3.59 (1H, m), 3.53 (3H, m), 3.45 (2H, m), 2.36 (2H, m); ^13^C NMR (150 MHz, Methanol-*d*_4_): *δ*_C_ 157.3 , 153.9 , 145.4, 138.9 , 137.4 , 136.8 , 135.3 , 134.5 , 132.2 , 128.2 , 126.9 , 126.9 , 124.4 , 122.6 , 118.4 , 118.0 , 118.0 , 103.0 , 78.2 , 77.9 , 75.0 , 74.7 , 71.4 , 62.6 , 45.8, 38.3.

1. †Jia-Ru Zhou and Xin-Yue Hu contributed equally to this work

   *Correspondence:

   [lixingren@mail.kib.ac.cn](mailto:lixingren@mail.kib.ac.cn) (X.-R. Li);

   [xugang008@mail.kib.ac.cn](mailto:xugang008@mail.kib.ac.cn) (G. Xu). [↑](#footnote-ref-1)
2. [↑](#footnote-ref-2)
